# Myeloid‐Derived Grancalcin Promotes Periodontal Inflammation and Pathological Bone Remodeling in Periodontitis

**DOI:** 10.1002/advs.202519267

**Published:** 2026-03-25

**Authors:** Min Zhou, Yao Xiao, Ziyue Xu, Chengyu Yang, Jiang Li, Ya Liu, Ping Yin

**Affiliations:** ^1^ Department of Endocrinology Endocrinology Research Center Xiangya Hospital of Central South University Changsha Hunan China; ^2^ Department of Oral and Maxillofacial Surgery Center of Stomatology Xiangya Hospital Central South University Changsha Hunan China

**Keywords:** grancalcin, hydrogel therapy, inflammation, myeloid cells, osteoclastogenesis, periodontitis

## Abstract

Periodontitis is a common chronic oral inflammatory disease characterized by enhanced inflammation and alveolar bone loss, yet its underlying mechanisms remain incompletely understood. Here, we identified grancalcin (GCA), a protein secreted by myeloid‐derived cells, as significantly enriched in the gingival tissues of both human patients and mouse models of periodontitis. Genetic ablation of GCA, either through global knockout or myeloid‐specific conditional deletion, markedly alleviated periodontal inflammation and pathological bone remodeling. Mechanistically, in primary human gingival fibroblasts, GCA interacts with CD44, which subsequently activates nonmuscle myosin heavy chain IIA (MYH9). MYH9 drives NF‐κB pathway activation and the consequent inflammatory response by promoting the nuclear translocation of its subunit p65, ultimately leading to exacerbated gingival inflammation and pathological bone resorption. Given that a GCA‐neutralizing antibody mitigated these pathogenic processes, we developed a thermosensitive hydroxybutyl chitosan hydrogel for its controlled local delivery, which successfully alleviated periodontitis. Collectively, our findings unveil GCA as a previously unrecognized regulator of periodontitis and highlight its potential as both a molecular marker and a therapeutic target for this disease.

## Graphical abstract

Grancalcin (GCA), a myeloid‐derived protein, is enriched in gingival tissues of periodontitis patients and mouse models. Through interactions with CD44 and activation of MYH9, GCA promotes NF‐κB signaling and exacerbates periodontal inflammation and bone loss. Targeting GCA with a neutralizing antibody using a thermosensitive hydrogel offers a promising strategy to alleviate periodontitis.

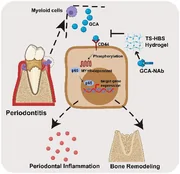

## 1 Introduction

Periodontitis is a prevalent oral inflammatory disease worldwide [1, 2, 3], characterized by the progressive destruction of periodontal connective tissue and alveolar bone [4]. It is primarily driven by a dysbiotic microbial biofilm that triggers a sustained and dysregulated host immune response, leading to persistent inflammation, tissue degradation, and bone resorption, ultimately resulting in tooth loss [5]. Beyond its local effects, periodontitis has been increasingly associated with several systemic diseases, including cardiovascular disease, diabetes, and rheumatoid arthritis [6, 7, 8, 9, 10, 11, 12, 13]. This substantial burden underscores the urgent need to further elucidate its pathogenesis and develop novel therapeutic strategies.

Immune cells orchestrate the initiation and progression of periodontitis via a multitude of mechanisms [14]. Proinflammatory macrophages and T cells, which accumulate in gingival tissue during periodontitis, induce the expression of receptor activator of nuclear factor‐κB ligand (RANKL), promote osteoclastogenesis, and subsequently drive alveolar bone loss [15, 16, 17, 18]. Both impaired and excessive neutrophil responses can exacerbate the local inflammatory response and promote matrix degradation [19, 20, 21]. Furthermore, immune cells can induce fibroblasts to produce cytokines and matrix metalloproteinases, leading to the disruption of tissue structure [22, 23, 24, 25, 26]. These findings highlight the complex and dynamic roles of immune cells in periodontitis, and understanding these mechanisms is essential for developing targeted therapies and preserving periodontal health.

Grancalcin (GCA), an EF‐hand Ca^2^^+^‐binding protein, is a member of the S100/calgranulin superfamily [27, 28]. Unlike classical calgranulins such as S100A8 and S100A9, which are broadly expressed in neutrophils and monocytes, GCA exhibits a more restricted expression pattern, being enriched in proinflammatory and senescent subsets of both neutrophils and macrophages [29]. This distinct expression profile aligns with its emerging role as a critical regulator of aging‐related pathologies, such as the aggravation of bone loss, induction of adipose tissue inflammation and insulin resistance, and promotion of cognitive decline [29, 30, 31]. While these findings underscore its significance in systemic inflammatory and age‐related diseases, the specific role of GCA in periodontal inflammation and tissue destruction remains largely unexplored.

Our study identifies myeloid‐derived GCA as a newly discovered regulator that accumulates in diseased gingiva and drives periodontitis progression. Mechanistically, GCA engages gingival fibroblasts via the CD44/MYH9 axis to activate the NF‐κB pathway, fueling inflammation and bone loss. Targeting this axis, we developed a GCA‐neutralizing antibody (GCA‐NAb) and encapsulated it within a thermosensitive hydrogel for localized delivery. This targeted strategy effectively attenuated both inflammatory and bone‐destructive phenotypes in vivo. These results collectively establish GCA as a key contributor to periodontitis and a candidate for targeted intervention.

## 2 Results

### 2.1 GCA Accumulates in the Gingiva of Periodontitis Patients and Mice

**FIGURE 1:**
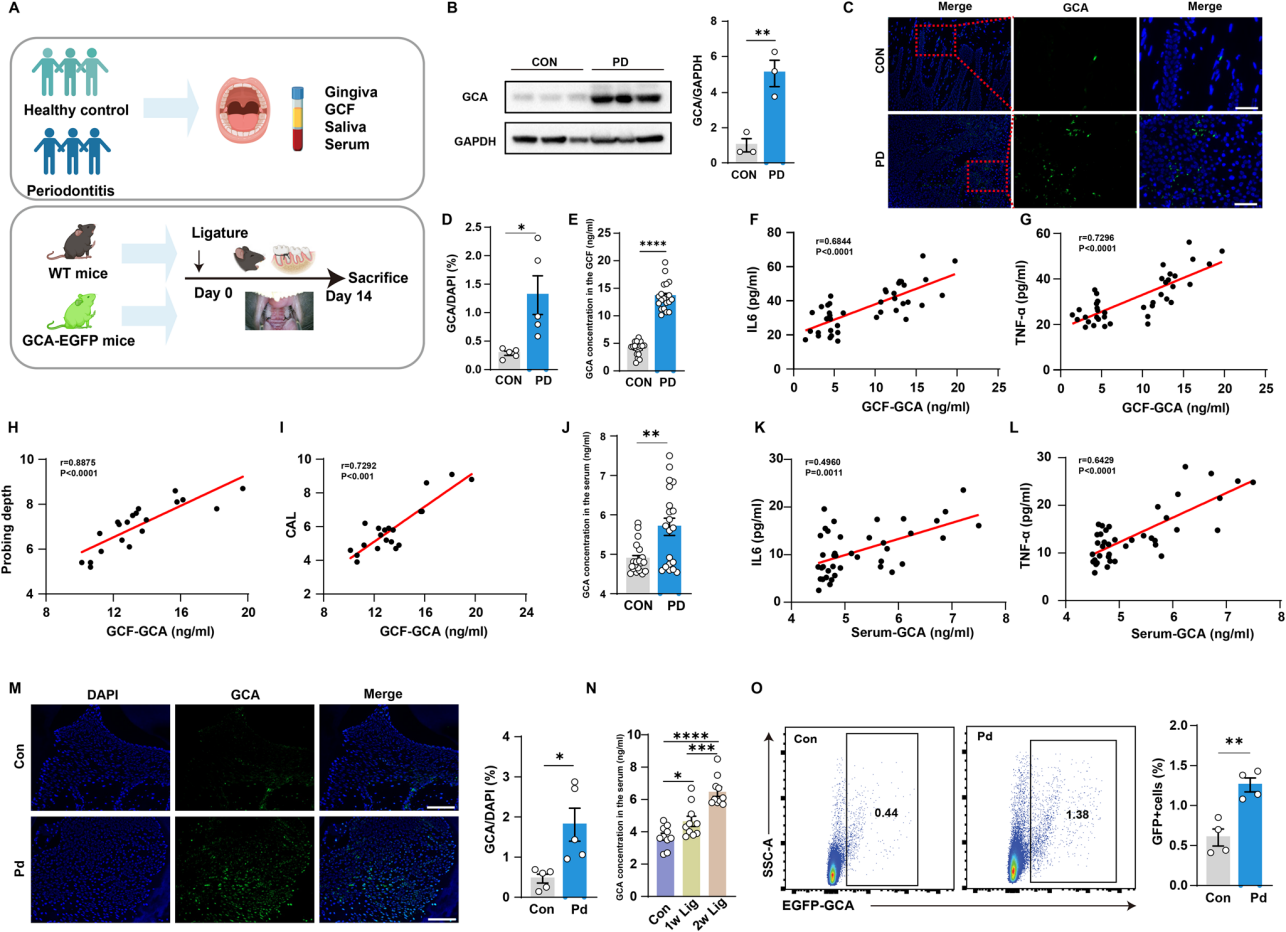
GCA accumulates in the gingiva of periodontitis patients and mice. (A) Schematic illustration of the experimental design and workflow. Created with BioRender.com. (B) Representative western blot images and quantitative analysis of GCA protein expression in gingiva from controls (CON) and periodontitis (PD) individuals (*n* = 3). (C,D) Representative immunofluorescence staining (C) and quantitative analysis (D) of GCA (green) in gingiva from CON and PD individuals (scale bars, 40 µm; *n* = 5). (E) GCA concentration in the gingival crevicular fluid (GCF) from CON and PD individuals (*n* = 20). (F,G) Correlation analysis between GCA and interleukin‐6 (IL‐6, F) or tumor necrosis factor‐α (TNF‐α, G) in GCF from human participants (*n* = 40). (H,I) Correlation analysis between GCF GCA and probing depth or clinical attachment loss (CAL) from PD participants (*n* = 20). (J) Serum GCA concentrations in CON and PD individuals (*n* = 20). (K,L) Correlation analysis between serum GCA and IL‐6 (K) or TNF‐α (L) from human participants (*n* = 40). (M) Representative immunofluorescence staining and quantitative analysis of GCA (green) in gingiva from control (Con) and periodontitis (Pd) mice (scale bars, 100 µm; *n* = 5). (N) Serum GCA concentrations in Con, one‐week ligature (1w Lig), and two‐week ligature (2w Lig) mice (*n* = 10). (O) Representative flow cytometry plots and quantification of GCA expression in gingiva from Con and Pd Gca‐Cre‐EGFP mice (*n* = 4). Data are presented as mean ± SEM. *n* indicates the number of biologically independent samples examined. Statistical significance was determined by two‐sided Student's *t*‐test (B,D,E,J,M,O), one‐way ANOVA with Tukey's multiple‐comparison test (N), or two‐sided Spearman's correlation analysis (F–I,K,L). *p < 0.05, **p < 0.01, ***p < 0.001, ****p < 0.0001.

To explore the relationship between GCA level and periodontitis, we first examined its expression in human gingival tissues. We recruited 20 patients with periodontitis (PD) and 20 systemically healthy controls (CON), all of whom were nonsmokers and free from systemic diseases such as diabetes or conditions that could influence periodontal status (Figure 1A). A significant increase in GCA was observed within the PD gingival samples at both mRNA and protein level (Figure S1A and Figure 1B). Immunofluorescence and immunohistochemistry validated these findings (Figure 1C,D and Figure S1B). Furthermore, analysis of two representative noninvasive oral biofluids, gingival crevicular fluid (GCF) and saliva, revealed markedly increased GCA levels in PD patients (Figure 1E and Figure S1C). Notably, GCA levels in both GCF and saliva showed positive correlations with local proinflammatory cytokines (interleukin‐6 [IL‐6] and tumor necrosis factor‐α [TNF‐α]) and key clinical parameters of disease severity, including probing depth and clinical attachment loss (CAL) (Figure 1F–I and Figure S1D–G). We further extended our analysis to systemic circulation and similarly observed a significant increase in blood GCA levels in PD patients (Figure 1J), which also correlated positively with serum levels of IL‐6 and TNF‐α (Figure 1K,L). To evaluate the diagnostic potential of circulating GCA, we performed receiver operating characteristic (ROC) curve analysis. The result demonstrated an area under the curve (AUC) of 0.731 for serum GCA in distinguishing periodontitis patients from healthy controls (Figure S1H), suggesting its potential diagnostic capacity. Collectively, these findings establish a robust association between elevated GCA levels and both local and systemic inflammatory burden in periodontitis, highlighting GCA as a potential indicator of periodontitis.

We next examined GCA expression in a mouse model of ligature‐induced periodontitis. After two weeks of ligation, when the periodontitis model is fully established (Figure S1I), increased GCA accumulation was detected in gingival tissues (Figure S1J,K and Figure 1M). Consistent with this local increase, serum GCA levels were also significantly elevated, showing a rise after one week of ligation and a further increase after two weeks (Figure 1N). To directly visualize the distribution of GCA, we generated *Gca*‐Cre‐EGFP reporter mice (Figure S1L,M), in which the EGFP signal indicates GCA localization. Flow cytometry analysis confirmed an enhanced level of GCA in periodontal tissues of ligatured reporter mice (Figure 1O).

Taken together, these results establish that GCA accumulation in gingival tissues is a consistent feature of periodontitis, observed across both human disease and mouse models.

### 2.2 Genetic Deletion of *Gca* Mitigates Periodontal Pathology in Mice with Periodontitis

**FIGURE 2:**
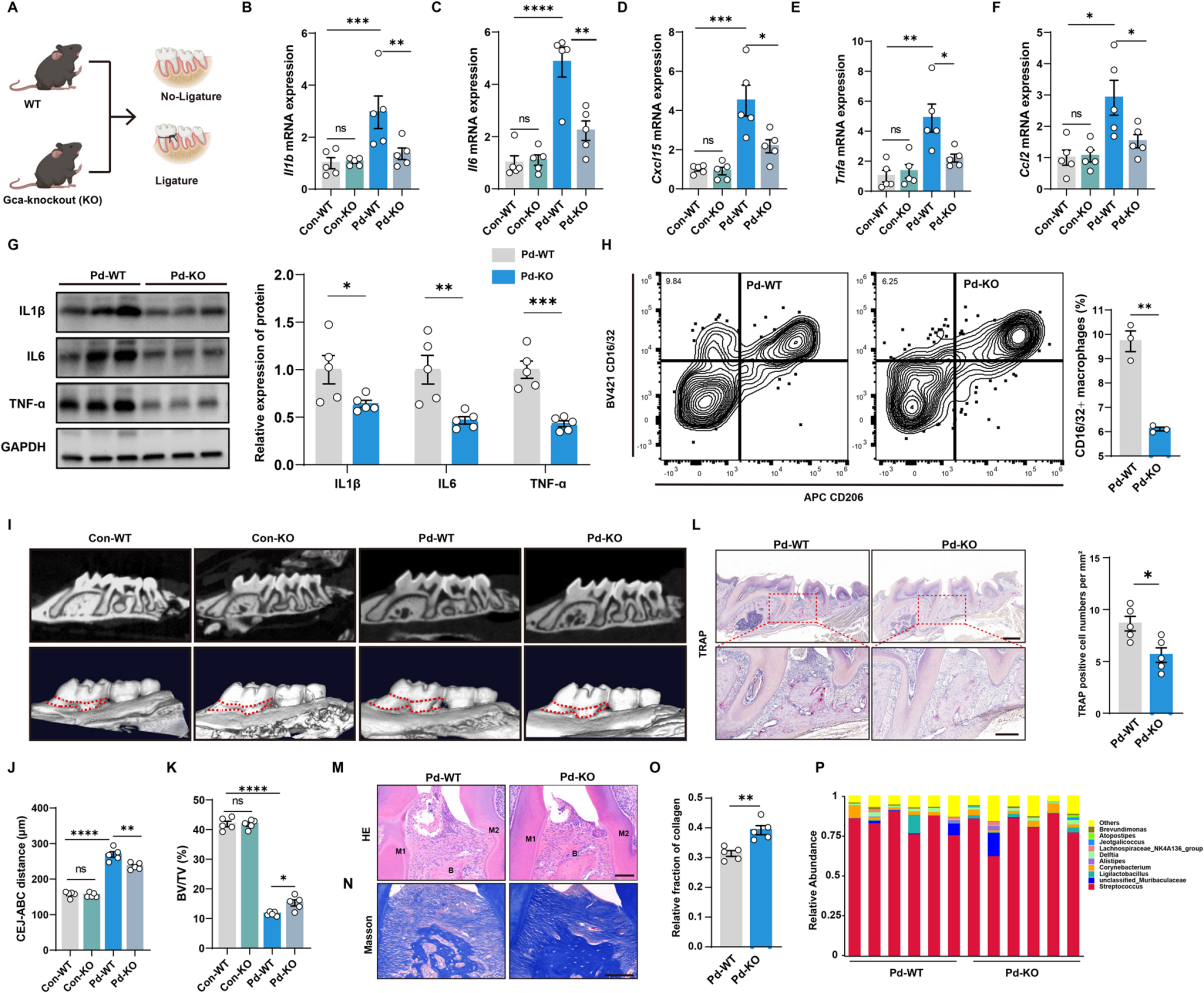
Genetic deletion of *Gca* mitigates periodontal pathology in mice with periodontitis. (A) Schematic illustration of ligature‐induced periodontitis models in wild‐type (WT) and *Gca*‐knockout (KO) mice. Created with BioRender.com. (B–F) Relative mRNA expression of proinflammatory markers in gingival tissues from WT and *Gca*‐KO mice under control or ligature conditions (*n* = 5). (G) Representative images (left) and quantification (right) of western blot analysis showing protein expression of IL‐1β, IL‐6, and TNF‐α in gingival tissues from ligatured WT and *Gca*‐KO mice (*n* = 5). (H) Flow cytometric analysis (left) and quantification (right) of CD16/32^+^ proinflammatory macrophages in gingival tissues from ligatured WT and *Gca*‐KO mice (gating strategy shown in Figure S2B; *n* = 3). (I) Representative micro‐CT images and 3D reconstructions of maxillae from WT and *Gca*‐KO mice under control or ligature conditions. (J,K) Quantitative analysis of the cement‐to‐enamel junction to alveolar bone crest (CEJ–ABC) distance (J) and bone volume to total volume (BV/TV) (K) based on micro‐CT images (*n* = 5). (L) Representative TRAP staining (left) and quantification (right) of TRAP‐positive cells per millimeter of alveolar bone surface in ligatured WT and *Gca*‐KO mice (scale bars, 500 µm (upper) or 200 µm (lower); *n* = 5). (M) Representative H&E staining of periodontal tissues from WT and *Gca*‐KO mice (scale bars, 100 µm). M1, the first upper molars; M2, the second upper molars; B, interdental alveolar bone. (N,O) Representative Masson's trichrome staining (N) and quantification (O) of collagen volume fraction in gingival tissues based on Masson**'s trichrome** staining (scale bars, 100 µm; *n* = 5). (P) Relative abundance of major bacterial genera in gingival samples from ligatured WT and *Gca*‐KO mice determined by 16S rRNA gene sequencing (*n* = 6). Data are presented as mean ± SEM. *n* indicates the number of biologically independent samples examined. Statistical significance was determined by two‐sided Student's *t*‐test (G,H,L,O) or two‐way ANOVA with Tukey's multiple‐comparison test (B–F,J–K). *p < 0.05, **p < 0.01, ***p < 0.001, ****p < 0.0001.

Given the observed association between GCA and periodontitis, we next assessed its contribution to the disease using global *Gca*‐knockout (KO) mice (Figure 2A and Figure S2A). Under physiological conditions, 8‐week‐old *Gca*‐KO mice displayed low inflammatory levels and alveolar bone volume similar to those of wild‐type (WT) mice, establishing a comparable baseline for subsequent comparisons (Figure 2B–F and Figure 2I–K). Following ligature‐induced periodontitis, inflammatory responses were markedly enhanced (Figure 2B–F); notably, *Gca*‐KO mice exhibited significantly lower mRNA and protein levels of proinflammatory markers in gingival tissues compared to WT controls (Figure 2B–G). Consistently, flow cytometric analysis revealed a marked reduction in CD16/32^+^ proinflammatory macrophages within gingival tissues of *Gca*‐KO mice relative to WT counterparts (Figure 2H). The gating strategy is shown in Figure S2B. Micro‐CT analysis demonstrated that alveolar bone structures were better preserved in *Gca*‐KO mice compared with WT mice under ligature‐induced periodontitis (Figure 2I–K). Moreover, *Gca*‐KO mice had fewer multinucleated osteoclasts within alveolar bone as demonstrated by TRAP staining (Figure 2L). Hematoxylin–eosin (H&E) staining further supported improved alveolar bone architecture (Figure 2M). Masson's trichrome staining revealed that gingival collagen fibers were maintained to a greater extent in *Gca‐*KO mice relative to WT mice under periodontitis conditions (Figure 2N,O). Importantly, comparative 16S rRNA gene sequencing analysis revealed no significant differences in overall microbial diversity or community structure between gingival samples from WT and *Gca*‐KO mice (Figure 2P and Figure S2C–G). These results suggest that gross alterations in the local microbiota are unlikely to account for the observed phenotypic differences. Collectively, these findings indicate that GCA deficiency alleviates periodontal pathology under ligature‐induced conditions by dampening inflammatory responses and mitigating pathological bone remodeling in periodontal tissues.

### 2.3 Myeloid‐Derived GCA Drives Proinflammatory and Osteoclastogenic Responses in Gingiva

**FIGURE 3:**
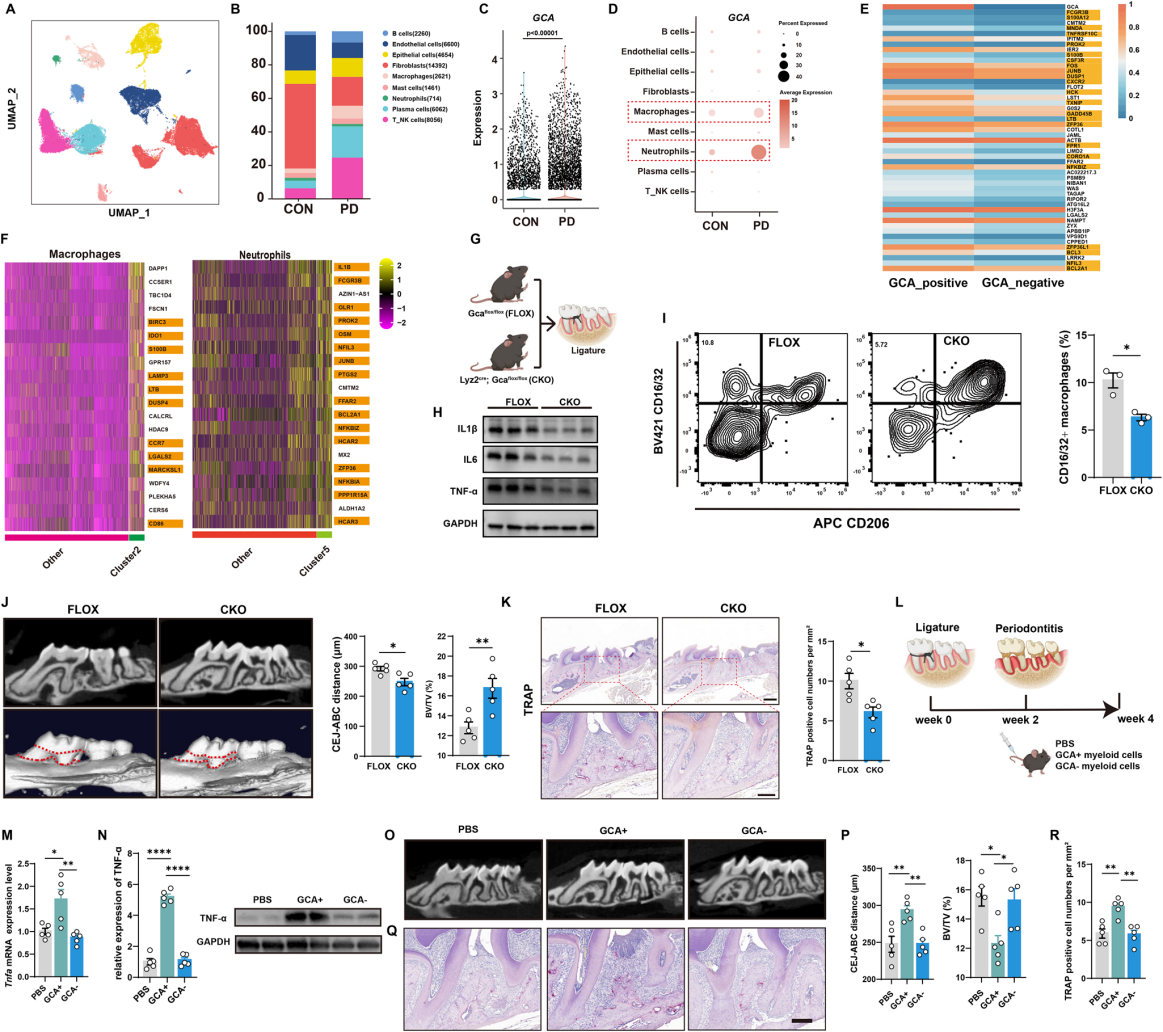
Myeloid‐derived GCA drives proinflammatory and osteoclastogenic responses in gingiva. (A,B) UMAP visualization (A) and proportional distribution of major cell types (B) of single‐cell RNA sequencing (scRNA‐seq) data from gingival tissues of control (CON) and periodontitis (PD) individuals. (C) Violin plot showing GCA expression levels in gingival tissues of CON and PD group. (D) Dot plot of GCA expression across annotated cell populations. (E) Heatmap of the top 50 upregulated genes in GCA^+^ versus GCA^−^ myeloid cells. (F) Heatmap of the top 20 upregulated genes in the macrophage_2 cluster (left) and neutrophil_5 cluster (right). (G) Schematic illustration of the ligature‐induced periodontitis model in GCA^flox/flox^ (FLOX) and Lyz2^Cre^; GCA^flox/flox^ (CKO) mice. Created with BioRender.com. (H) Representative western blot images showing protein expression of IL‐1β, IL‐6, and TNF‐α in gingival tissues from FLOX and CKO mice (*n* = 3). (I) Flow cytometric analysis and quantification of CD16/32^+^ proinflammatory macrophages in gingival tissues from FLOX and CKO mice (*n* = 3). (J) Representative micro‐CT images and 3D reconstructions of maxillae and quantitative analysis of alveolar bone loss, including the cement‐to‐enamel junction to alveolar bone crest (CEJ–ABC) distance and bone volume to total volume (BV/TV) (*n* = 5). (K) Representative TRAP staining of alveolar bone sections (left) and quantification (right) of TRAP‐positive cells per millimeter of alveolar bone surface (scale bars, 500 µm (upper) or 200 µm (lower); *n* = 5). (L) Schematic illustration of the transplantation model using FACS‐sorted EGFP^−^ and EGFP^+^ myeloid cells isolated from Gca‐Cre‐EGFP reporter mice, representing GCA^−^ and GCA^+^ myeloid populations, respectively. (M) Relative mRNA expression of *Tnfa* in gingival tissues from mice transplanted with GCA^−^ or GCA^+^ myeloid cells (*n* = 5). (N) Representative western blot images (right) and its quantification (left) showing TNF‐α protein expression in gingival tissues (*n* = 5). (O,P) Representative micro‐CT images (O) and quantitative analysis (P) of CEJ–ABC distance and BV/TV (*n* = 5). (Q,R) Representative TRAP staining (Q) of alveolar bone sections and quantification (R) of TRAP‐positive cells per millimeter of alveolar bone surface (scale bars, 200 µm; *n* = 5). Data are presented as mean ± SEM. *n* indicates the number of biologically independent samples examined. Statistical significance was determined by two‐sided Student's *t*‐test (I,J,K), one‐way ANOVA with Tukey's multiple‐comparison test (M,N,P,R). *p < 0.05, **p < 0.01, ***p < 0.001, ****p < 0.0001.

To identify the major cellular sources of GCA within periodontal tissues, we performed single‐cell RNA sequencing (scRNA‐seq) on gingival tissues from CON and PD individuals. This analysis revealed diverse immune and stromal cell populations (Figure 3A,B). Violin plots demonstrated a significant elevation of GCA expression in PD gingiva compared with healthy controls (Figure 3C). Dot plot analysis revealed that GCA expression was predominantly confined to myeloid‐derived clusters, particularly macrophages and neutrophils, whereas fibroblasts, endothelial cells, epithelial cells, mast cells, and lymphoid lineages (B cells, T cells, plasma cells) showed minimal or no detectable expression (Figure 3D). Accordingly, macrophages and neutrophils were collectively referred to as myeloid cells when assessing overall expression patterns. Compared with GCA^−^ myeloid cells, their GCA^+^ counterparts displayed significantly higher expression of inflammation‐ and immunity‐associated genes (Figure 3E).

To further delineate these subsets, single‐cell subclustering identified nine macrophage clusters and six neutrophil clusters within gingival tissues. Notably, the macrophage_2 and neutrophil_5 clusters exhibited the highest levels of GCA expression in PD samples (Figure S3A). Transcriptional profiling of these two clusters revealed enrichment of a broad spectrum of proinflammatory genes, including *BIRC3*, *IDO1*, *S100B*, *LAMP3*, *LTB*, *DUSP4*, *CCR7*, *LGALS2*, *MARCKSL1*, *CD16/32*, *IL1B*, *FCGR3B*, *OLR1*, *PROK2*, *OSM*, *NFIL3*, *JUNB*, *PTGS2*, *FFAR2*, *BCL2A1*, *NFKBIZ*, *HCAR2*, *ZFP36*, *NFKBIA*, *PPP1R15A*, and *HCAR3* (Figure 3F), indicating that GCA^+^ macrophages and neutrophils exhibit a pronounced proinflammatory transcriptional signature.

We further validated GCA expression in gingival tissues by immunofluorescence analysis. Given that macrophages constitute a major myeloid cell population (Figure 3B), this assessment revealed enhanced GCA expression and increased macrophage infiltration in periodontitis (Pd) gingival tissues, accompanied by a marked enrichment of GCA‐positive macrophages (Figure S3B). These findings further indicate the local accumulation of GCA‐expressing myeloid cells in periodontal lesions.

To investigate the role of myeloid‐derived GCA in the progression of periodontitis, *Gca* ^flox/flox^ mice (Figure S3C) were crossed with Lyz2‐Cre mice to generate myeloid‐specific *Gca* conditional knockout (*Gca*‐CKO) mice. The efficient deletion of *Gca* was confirmed in myeloid lineage cells (Figure S3D), and a corresponding reduction of GCA was also observed in the periodontium (Figure S3E). In ligature‐induced periodontitis models (Figure 3G), *Gca*‐CKO mice showed lower gingival mRNA and protein expression of proinflammatory mediators compared with littermate *Gca*‐FLOX controls (Figure S3F and Figure 3H). Flow cytometry further revealed a marked decrease in gingival CD16/32^+^ proinflammatory macrophages in *Gca*‐CKO mice (Figure 3I). Structurally, *Gca*‐CKO mice preserved more alveolar bone volume and had fewer TRAP‐positive multinucleated osteoclasts relative to FLOX controls (Figure 3J,K). H&E and Masson's trichrome staining also indicated improved preservation of alveolar bone levels and collagen fibers in CKO mice (Figure S3G,H).

To establish a direct causal role, we performed adoptive transfer experiments. GCA^+^ and GCA^−^ myeloid cells were isolated from periodontitis *Gca*‐Cre‐EGFP reporter mice and transferred into ligature‐induced *Gca*‐CKO recipients (Figure 3L). Strikingly, adoptive transfer of GCA^+^ myeloid cells substantially recapitulated key features of periodontitis, including gingival inflammation, osteoclastogenesis, and alveolar bone loss. Recipients of GCA^−^ cell retained a phenotype comparable to untransferred *Gca*‐CKO controls (Figure 3M–R).

Collectively, our findings define a key pathogenic mechanism in periodontitis, in which GCA, predominantly expressed by proinflammatory myeloid cells in the periodontal niche, critically drives local inflammation and osteoclastogenesis, leading to tissue destruction.

### 2.4 GCA Reprograms Gingival Fibroblasts to Amplify Periodontal Inflammation and Pathological Bone Remodeling

Given that GCA deficiency in mice resulted in reduced osteoclastogenesis and bone loss in vivo (Figures 2 and 3), we hypothesized that GCA might directly promote osteoclast differentiation. To test this, we treated bone marrow‐derived macrophages (BMMs) with recombinant GCA (rGCA). Unexpectedly, rGCA directly inhibited osteoclast differentiation (Figure S4A,B), consistent with previous reports demonstrating that rGCA directly suppresses BMM activity [29]. This apparent discrepancy prompted us to investigate whether additional cell types might contribute to the regulation of osteoclast differentiation in this context.

**FIGURE 4:**
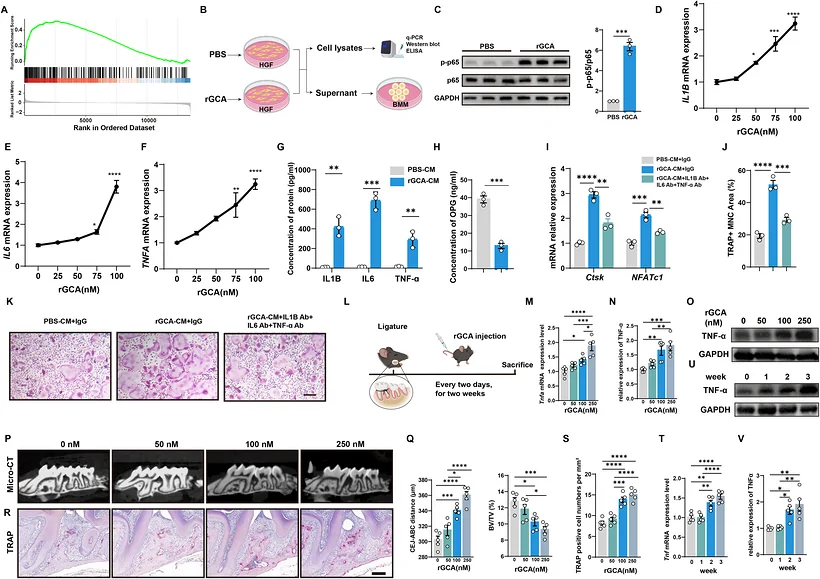
GCA drives gingival fibroblast dysfunction and exacerbates periodontal inflammation and pathological bone remodeling. (A) Gene set enrichment analysis (GSEA) of NF‐κB‐associated inflammatory gene signatures based on RNA‐seq data from recombinant GCA (rGCA)‐treated human gingival fibroblasts (HGFs). (B) Schematic illustration of the cell experimental workflow. Created with BioRender.com. (C) Representative western blot images (left) of phospho‐p65 (p‐p65) and total p65 protein and quantification (right) of the p‐p65/p65 ratio in HGFs treated with rGCA (*n* = 3). (D–F) RT‐qPCR analysis of proinflammatory cytokines in HGFs treated with rGCA at indicated concentrations (*n* = 3). (G,H) ELISA measurement of inflammatory cytokines (IL1β, IL6, and TNF‐α, G) and osteoprotegerin (OPG, H) in the culture supernatants (conditioned medium, CM) of periodontitis‐derived primary human gingival fibroblasts (PD‐HGFs) following stimulation with recombinant GCA (rGCA‐CM) or PBS control (PBS‐CM) (*n* = 3). (I–K) Bone marrow macrophages (BMMs) were cultured with macrophage colony‐stimulating factor (M‐CSF) and receptor activator of NF‐κB ligand (RANKL), and treated with PBS‐CM or rGCA‐CM. Osteoclast‐associated gene expression (Ctsk and Nfatc1) was quantified by RT‐qPCR (I). Representative TRAP staining images (K; scale bars, 200 µm) and quantitative analysis of osteoclast formation based on TRAP‐positive multinucleated cells (MNCs) area (%) (J) are shown (*n* = 3). (L) Schematic illustration of tail vein injection of graded doses of rGCA (0, 50, 100, 250 nm in 100 µL PBS, every other day for 2 weeks) in ligature‐induced periodontitis mice. Created with BioRender.com. (M–O) Relative mRNA expression of Tnfa (M) and representative western blot images with quantification of TNF‐α protein levels (N,O) in gingival tissues (*n* = 5). (P–S) Representative micro‐CT images and 3D reconstruction of maxillae (P), quantitative analysis of CEJ–ABC distance and BV/TV (Q), representative TRAP staining (R), and quantification of TRAP‐positive osteoclast numbers (S) (scale bars, 200 µm; *n* = 5). (T,V) Ligature‐induced periodontitis was first established for 2 weeks. After ligature removal, mice received tail vein injections of rGCA (250 nm in 100 µL PBS) every other day for an additional 0, 1, 2, or 3 weeks. Relative Tnfa mRNA expression (T) and representative western blot images with quantification of TNF‐α protein levels (U,V) are shown (*n* = 5). Data are presented as mean ± SEM. *n* indicates the number of biologically independent samples examined. Statistical significance was determined using two‐sided Student's *t*‐test (C,G,H), one‐way ANOVA followed by Dunnett's test for comparison with the control group (0 nm) (D–F) or Tukey's multiple‐comparison test (I,J,M,N,Q,S,T,V). *p < 0.05, **p < 0.01, ***p < 0.001, ****p < 0.0001.

Given that gingival fibroblasts (GFs) are the predominant stromal cell population in the gingiva and play a fundamental role in regulating inflammation and bone remodeling during periodontitis [22, 26, 32], we further investigated the role of GFs in periodontitis. Notably, our single‐cell RNA‐sequencing analysis revealed that upregulated genes in fibroblasts from PD patients were significantly enriched in the TNF‐α signaling via NF‐κB (Figure S4C). We further performed RNA sequencing (RNA‐seq) on isolated and validated human gingival fibroblasts (HGFs) treated with rGCA (Figure S4D). Gene set enrichment analysis demonstrated significant enrichment of rGCA‐regulated genes in the NF‐κB signaling pathway (Figure 4A). To confirm the proinflammatory effects of rGCA, we treated HGFs with rGCA (Figure 4B) and observed a robust increase in phosphorylation of the NF‐κB p65 subunit (Figure 4C). rGCA treatment also induced a dose‐dependent upregulation of proinflammatory cytokines at the transcriptional level, including *IL1B*, *IL6*, *TNFA*, *CXCL8*, and *CCL2*, which are canonical downstream targets of NF‐κB signaling (Figure 4D–F and Figure S4F,G); this was accompanied by the downregulation of osteoclastogenesis‐inhibitory factors, including *IL4* and *TNFRSF11B* (Figure S4H,I). Meanwhile, these changes occurred without any effect on cell viability (Figure S4E).

Furthermore, we analyzed the conditioned medium from primary periodontitis‐derived human gingival fibroblasts (PD‐HGFs) treated with rGCA. We observed a marked increase in proinflammatory cytokines, including IL1β, IL6, and TNF‐α (Figure 4G), accompanied by a significant reduction in osteoprotegerin (OPG) levels (Figure 4H) in the conditioned medium. Strikingly, conditioned medium from rGCA‐treated PD‐HGFs robustly enhanced BMM osteoclastogenesis (Figure 4I–K). When a neutralizing antibody cocktail against IL1β, IL6, and TNF‐α was added during rGCA treatment of PD‐HGFs, the pro‐osteoclastogenic effect was markedly attenuated (Figure 4I–K). These findings indicate that GCA‐induced fibroblast inflammation and the subsequent release of proinflammatory cytokines mediate the enhanced osteoclastogenic response.

To determine whether these observations could be recapitulated in vivo, we administered rGCA at increasing concentrations (0, 50, 100, and 250 nm in 100 µL phosphate‐buffered saline [PBS]) via tail vein injection every other day for two weeks to mice with ligature‐induced periodontitis (Figure 4L). We found that higher concentrations of rGCA led to progressively increased TNF‐α expression in the gingiva and significantly exacerbated alveolar bone loss (Figure 4M–S). Flow cytometry further revealed a significant expansion of CD16/32^+^ proinflammatory macrophages in the gingiva of rGCA‐treated mice at the 250 nm dose (Figure S4J,K). H&E and Masson's trichrome staining confirmed reduced preservation of alveolar bone and collagen fibers in the 250 nm rGCA group compared to controls (Figure S4L,M). In a separate time–course experiment, mice with periodontitis were treated with 250 nm rGCA and tissues were harvested at different time points (Figure S4N). This experiment demonstrated that the extent of gingival inflammation and bone loss worsened with the duration of rGCA administration (Figure 4T–V and Figure S4O–Q). These findings demonstrate that exogenous GCA exacerbates periodontitis in a dose‐ and time‐dependent manner.

In summary, our data establish GCA as a critical driver of periodontitis. Mechanistically, rather than acting directly on osteoclast precursors, GCA induces a proinflammatory reprogramming of gingival fibroblasts, leading to enhanced secretion of inflammatory cytokines and suppression of osteoclast‐inhibitory factors, thereby amplifying local inflammation and pathological bone remodeling.

### 2.5 GCA Promotes Inflammation via the CD44–NF‐κB Signaling Pathway in HGFs

**FIGURE 5:**
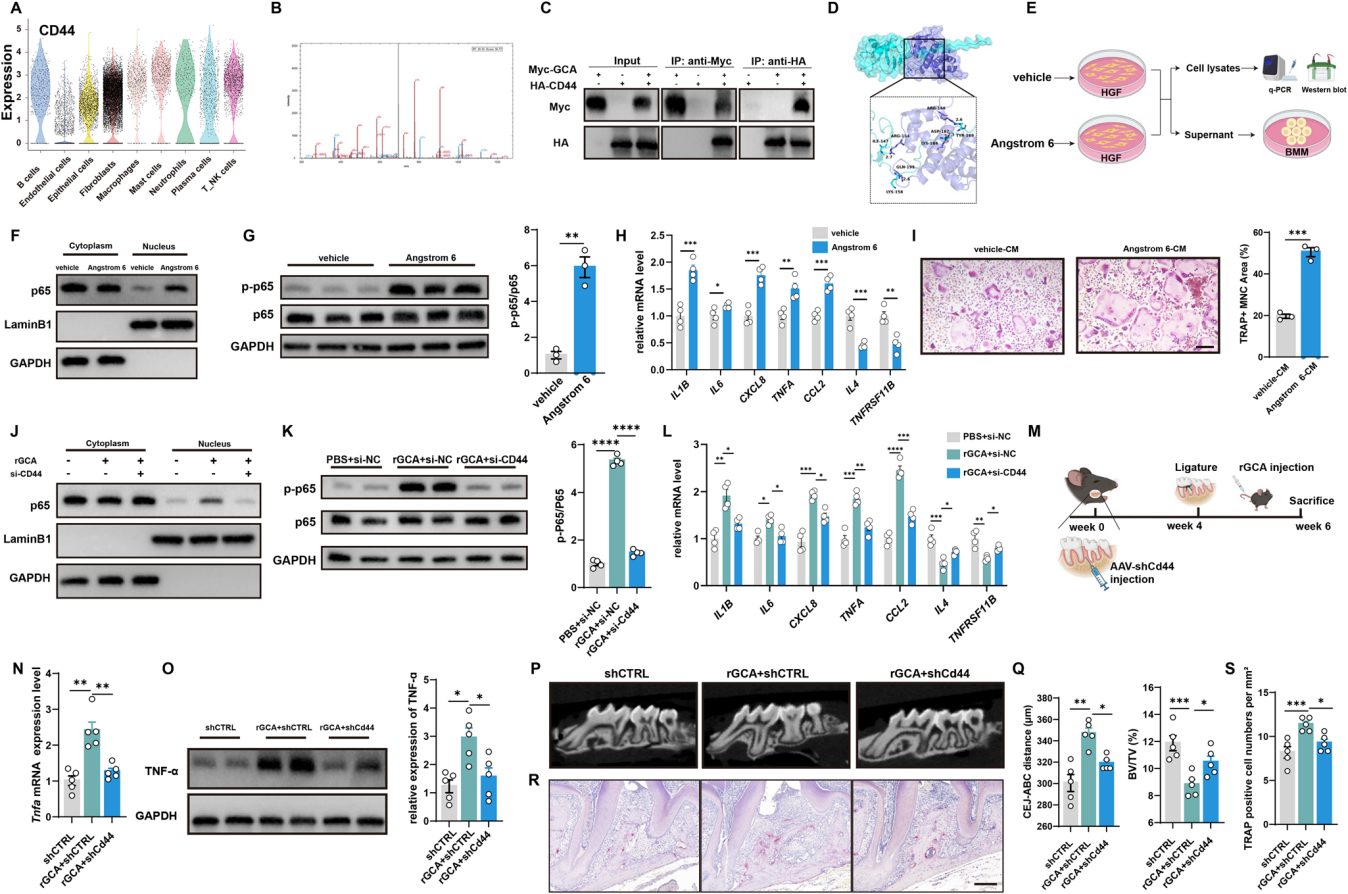
GCA promotes inflammation via the CD44–NF‐κB signaling pathway in HGFs. (A) Violin plots showing *CD44* expression across gingival cell types in scRNA‐seq data. (B) Secondary mass spectrometry spectrum showing the fragmentation pattern of a CD44 peptide detected in rGCA immunoprecipitates. (C) Co‐immunoprecipitation confirming the interaction between Myc‐GCA and HA‐CD44. (D) Molecular docking model illustrating the GCA–CD44 interface. GCA was displayed as a slate cartoon model, CD44 as a cyan cartoon model, and binding sites were highlighted as stick representations in corresponding colors. (E) Schematic illustration of the cell experimental workflow. Created with BioRender.com. (F) Representative western blot images of p65 in cytoplasmic and nuclear lysates from human gingival fibroblasts (HGFs) treated with Angstrom 6 or vehicle. (G) Representative western blot images showing phosphorylated p65 (p‐p65) and total p65 in HGFs, with quantification of the p‐p65/p65 ratio (*n* = 3). (H) RT‐qPCR analysis of proinflammatory cytokines and osteoclast‐inhibitory factors (*n* = 4). (I) Bone marrow macrophages (BMMs) were cultured with macrophage colony‐stimulating factor (M‐CSF) and receptor activator of NF‐κB ligand (RANKL), and treated with conditioned medium (CM) from PD‐HGFs pretreated with vehicle (vehicle‐CM) or Angstrom 6 (Angstrom 6‐CM). Representative TRAP staining and quantification of TRAP‐positive multinucleated cells (MNCs) area (%) are shown (scale bars, 200 µm; *n* = 3). (J) Representative western blot images of p65 in cytoplasmic and nuclear lysates from HGFs transfected with siRNA‐NC (si‐NC) and siRNA‐CD44 (si‐CD44), with or without rGCA treatment. (K) Representative western blot images showing phosphorylated p65 (p‐p65) and total p65 in HGFs, with quantification of the p‐p65/p65 ratio (*n* = 4). (L) RT‐qPCR analysis of proinflammatory cytokines and osteoclast‐inhibitory factors (*n* = 4). (M) In vivo experimental scheme: mice received periodontal injection of AAV‐shCTRL or AAV‐shCd44. Four weeks later, ligature‐induced periodontitis was initiated, followed by rGCA administration (250 nm in 100 µL PBS) via tail vein for 2 weeks. Created with BioRender.com. (N) Relative mRNA expression of *Tnfa* in gingival tissues (*n* = 5). (O) Representative western blot images (left) and its quantification (right) showing TNF‐α protein expression in gingival tissues (*n* = 5). (P,Q) Representative micro‐CT images (P) and quantitative analysis (Q) of the cement‐to‐enamel junction to alveolar bone crest (CEJ–ABC) distance and bone volume to total volume (BV/TV) (*n* = 5). (R,S) Representative TRAP staining of alveolar bone sections (R) and quantification (S) of TRAP‐positive cells per millimeter of alveolar bone surface (scale bars, 200 µm; *n* = 5). Data are shown as the mean ± SEM. *n* indicates the number of biologically independent samples examined. Statistical significance was determined using two‐sided Student's *t*‐test (G–I), one‐way ANOVA followed by Tukey's multiple‐comparison test (K,L,N,O,Q,S). *p < 0.05, **p < 0.01, ***p < 0.001, ****p < 0.0001.

Given the proinflammatory effect of GCA on fibroblasts, we next sought to identify its potential receptor. To this end, membrane proteins were extracted from HGFs treated with rGCA and analyzed by co‐immunoprecipitation followed by mass spectrometry (Co‐IP/MS). Among the proteins identified, four were previously reported as membrane receptors (Figure S4R). Of these, CD44 was found to be highly expressed in fibroblasts (Figure 5A,B and Figure S4S) and has been reported to play a critical role in the regulation of inflammatory signaling [33]. Co‐IP further confirmed the interaction between GCA and CD44 (Figure 5C). Furthermore, docking analysis with structural visualization in PyMOL predicted key interacting residues, including a hydrogen bond between ARG166 of GCA and TYR169 of CD44 (Figure 5D). These findings prompted us to further investigate the role of CD44 in this process.

To further define the functional role of CD44, we conducted a series of gain‐ and loss‐of‐function experiments in vitro (Figure 5E). Treatment with a known CD44 activator Angstrom 6 [34] robustly activated the NF‐κB signaling pathway, as evidenced by enhanced nuclear translocation and phosphorylation of NF‐κB p65 (Figure 5F,G). RT‐qPCR analysis revealed increased expression of multiple proinflammatory cytokines, accompanied by downregulation of *IL4* and *TNFRSF11B* (Figure 5H). Conditioned medium from CD44‐activated fibroblasts potently induced osteoclastogenesis in BMMs (Figure 5I), indicating that CD44‐induced secretory factors from fibroblasts are sufficient to promote osteoclast differentiation. Conversely, siRNA‐mediated knockdown of CD44 attenuated NF‐κB nuclear translocation and phosphorylation (Figure 5J,K), as well as the expression of proinflammatory cytokines in fibroblasts (Figure 5L). Increased *IL4* and *TNFRSF11B* mRNA expression were observed following CD44 inhibition (Figure 5L). Together, these functional data establish CD44 as a key mediator linking GCA stimulation to NF‐κB activation and subsequent pro‐osteoclastogenic effects in fibroblasts.

To substantiate these findings in vivo, we employed an adeno‐associated virus (AAV)‐mediated knockdown approach. Mice subjected to rGCA treatment received local gingival injections of AAV‐Col1a1‐sh*Cd44* to preferentially deplete CD44 in gingival fibroblasts, with AAV‐Col1a1‐shCtrl serving as the control (Figure 5M). In a ligature‐induced periodontitis model, mice with fibroblast‐specific *Cd44* knockdown showed reduced levels of inflammatory cytokines compared to control mice (Figure 5N,O). Moreover, CD44‐knockdown mice exhibited pronounced protection against periodontitis‐induced damage, as evidenced by greater preservation of alveolar bone volume and a lower number of TRAP‐positive osteoclasts compared to control mice under identical ligature conditions (Figure 5P–S).

### 2.6 The CD44–MYH9–NF‐κB Axis Mediates GCA‐Induced Inflammation During Peritonitis

**FIGURE 6:**
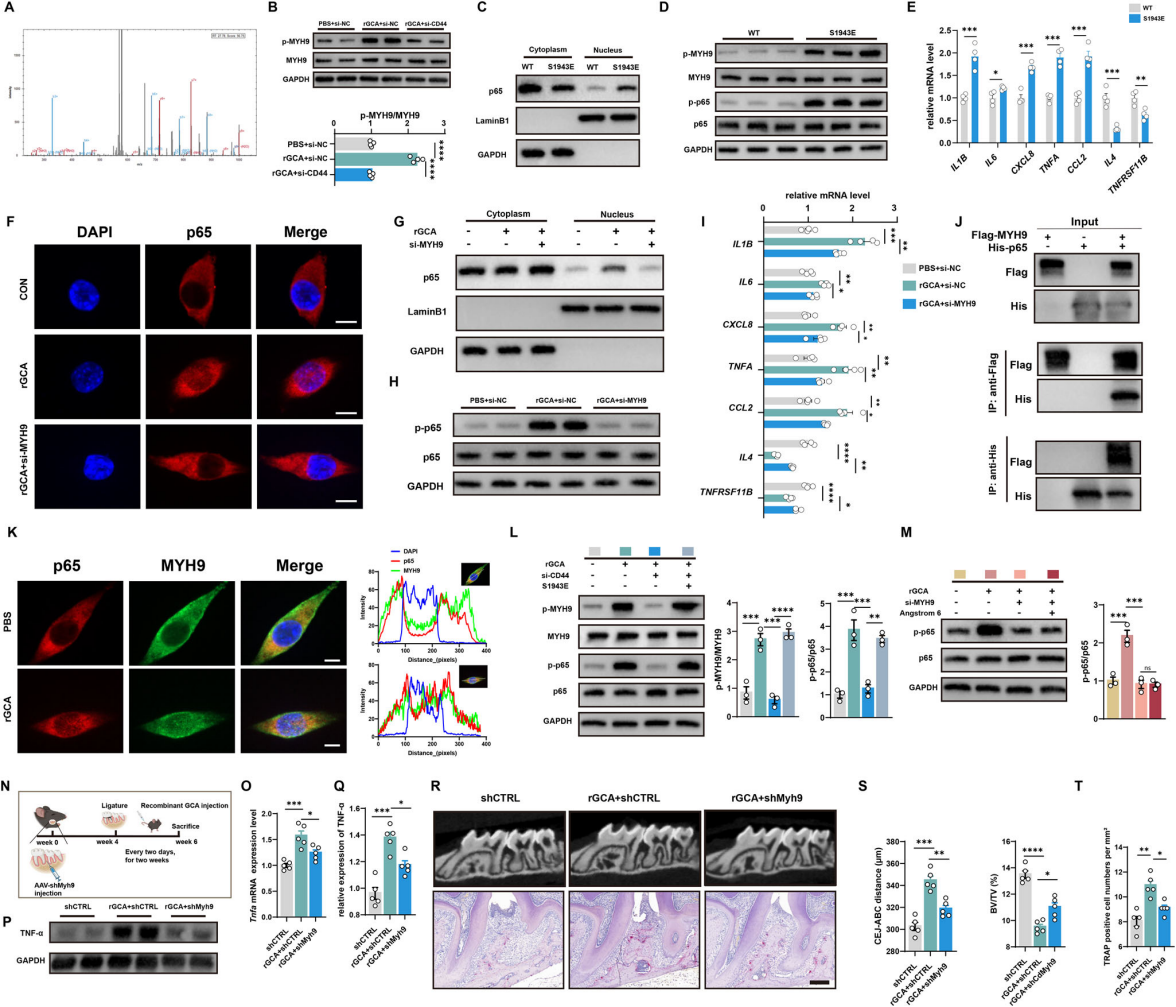
The CD44–MYH9–NF‐κB axis mediates GCA‐induced inflammation during periodontitis. (A) Representative MS/MS spectrum of an MYH9‐derived peptide identified from p65 immunoprecipitates in HGFs. (B) Representative western blot images (upper) showing phosphorylated MYH9 (p‐MYH9, Ser1943) and total MYH9, and quantitative analysis (lower) of the p‐MYH9/MYH9 ratio (*n* = 4). (C) Western blot analysis of p65 distribution in cytoplasmic and nuclear fractions in HGFs transfected with WT or S1943E MYH9. (D) Representative western blot images showing p‐MYH9 and total MYH9, and phosphorylated (p‐p65) and total p65 in HGFs treated as described in (C). (E) RT‐qPCR analysis of proinflammatory cytokines and osteoclast‐inhibitory factors (*n* = 4). (F) Immunofluorescence staining of p65 (red) in HGF cells transfected with siRNA‐NC (si‐NC) or siRNA‐MYH9 (si‐MYH9), treated with or without rGCA (scale bars, 10 µm). (G) Representative western blot images of p65 in cytoplasmic and nuclear lysates from HGFs treated as described in (F). (H) Representative western blot images showing p‐MYH9 and total MYH9, and p‐p65 and total p65 in HGFs treated as described in (F). (I) RT‐qPCR analysis of proinflammatory cytokines and osteoclast‐inhibitory factors in HGFs treated as described in (F) (*n* = 4). (J) Co‐immunoprecipitation (Co‐IP) confirming interaction between His‐p65 and Flag‐MYH9. (K) Immunofluorescence staining of p65 (red) and MYH9 (green) in HGFs treated with or without rGCA (scale bars, 10 µm). Corresponding line‐scan plots show fluorescence intensity profiles of p65 and MYH9 along the indicated line, quantified using ImageJ Plot Profile. (L) HGFs were transfected with siRNA‐NC (si‐NC) or siRNA‐CD44 (si‐CD44), or with MYH9 WT or MYH9 S1943E plasmids. Representative western blot images show phosphorylated MYH9 (p‐MYH9) and total MYH9, phosphorylated p65 (p‐p65) and total p65, along with quantitative analysis of the p‐MYH9/MYH9 and p‐p65/p65 ratios (*n* = 4). (M) HGFs were transfected with siRNA‐NC (si‐NC) or siRNA‐MYH9 (si‐MYH9), further treated with or without Angstrom 6. Representative western blot images show phosphorylated p65 (p‐p65) and total p65, along with quantitative analysis of the p‐p65/p65 ratios (*n* = 4). (N) Mice received periodontal injections of AAV‐CTRL or AAV‐shMyh9, and ligature‐induced periodontitis was initiated 4 weeks later, followed by rGCA administration (250 nm in 100 µL PBS) via tail vein for 2 weeks. Created with BioRender.com. (O) Relative mRNA expression of *Tnfa* in gingival tissues (*n* = 5). (P,Q) Representative western blot images (P) and quantification (Q) showing TNF‐α protein expression in gingival tissues (*n* = 5). (R) Representative micro‐CT images (upper) and TRAP staining (lower) of alveolar bone sections (scale bars, 200 µm). (S) Quantitative analysis of CEJ–ABC distance and BV/TV based on micro‐CT images (*n* = 5). (T) Quantification of TRAP‐positive cells per millimeter of alveolar bone surface (*n* = 5). Data are shown as the mean ± SEM. *n* indicates the number of biologically independent samples examined. Statistical significance was determined using two‐sided Student's *t*‐test (E), one‐way ANOVA followed by Tukey's multiple‐comparison test (B,I,L,M,O,Q,S,T). *p < 0.05, **p < 0.01, ***p < 0.001, ****p < 0.0001.

We next investigated the mechanism of NF‐κB activation by the GCA–CD44 axis. Notably, rGCA treatment induced robust nuclear translocation of the NF‐κB subunit p65 (Figure 5J), implying the involvement of additional molecular mediators. To identify p65‐interacting proteins under this condition, we performed Co‐IP/MS analysis using cells transfected with a His‐tagged p65 plasmid and stimulated with rGCA. Strikingly, MYH9, a cytoskeletal protein, ranked as the top candidate from our screen (Figure 6A).

Prior studies have established that MYH9 phosphorylation enables its function as a molecular motor, driving the nuclear translocation of the mitochondrial peptide MOTS‐c [35]. In this study, we asked whether the GCA–CD44 axis similarly activates MYH9. Indeed, GCA treatment robustly induced MYH9 phosphorylation, an effect abolished by CD44 inhibition (Figure 6B). To test whether phosphorylated MYH9 mediates p65 nuclear translocation and activation, we expressed a constitutively active MYH9 mutant in HGFs (S1943E). This enhanced MYH9 phosphorylation not only increased nuclear accumulation and phosphorylation of p65 but also upregulated inflammatory mediators while concomitantly downregulating osteoclast‐inhibitory genes (Figure 6C–E). Conversely, MYH9 knockdown attenuated rGCA‐induced p65 nuclear translocation, as evidenced by both immunofluorescence and western blot analyses (Figure 6F,G). It also suppressed p65 phosphorylation and the subsequent upregulation of inflammatory factors, while restoring the expression of osteoclast‐inhibitory genes (Figure 6H,I). Together, these data demonstrate that MYH9 activation is both necessary and sufficient for GCA‐triggered inflammation. Mechanistically, Co‐IP showed interaction between MYH9 and p65 (Figure 6J), which was further supported by immunofluorescence showing their pronounced colocalization in the nucleus following GCA stimulation (Figure 6K), indicating that MYH9 may function as a cytoskeletal regulator facilitating p65 nuclear accumulation, thereby promoting NF‐κB activation.

To further delineate the proinflammatory cascade of GCA–CD44–MYH9 in fibroblasts, we inhibited CD44 expression during rGCA treatment. This inhibition reduced p65 phosphorylation (Figure 6L), an effect that was significantly rescued by overexpressing the constitutively active MYH9 mutant (Figure 6L). In a complementary set of experiments, when MYH9 expression was suppressed, the exogenous activation of CD44 failed to significantly alter p65 phosphorylation (Figure 6M). Collectively, these findings demonstrate that the CD44–MYH9–NF‐κB axis critically mediates GCA‐driven inflammatory responses in fibroblasts.

To validate the MYH9‐dependent mechanism in vivo, we used an AAV‐mediated fibroblast‐targeted knockdown strategy targeting MYH9. rGCA‐treated mice received local gingival injections of AAV‐Col1a1‐sh*Myh9* or control virus (Figure 6N). In the ligature‐induced periodontitis model, MYH9 knockdown significantly reduced gingival proinflammatory cytokine levels (Figure 6O–Q). Consistently, *Myh9*‐deficient mice showed marked protection from periodontal pathology, including preservation of alveolar bone volume, reduced numbers of TRAP**‐positive** osteoclasts (Figure 6R–T). Together, these findings provide in vivo evidence that fibroblast MYH9 is a critical mediator of GCA‐driven inflammation and periodontal bone loss.

### 2.7 Systemic Neutralization of GCA Attenuates Inflammation and Preserves Periodontal Tissues and Bone

Given the role of GCA in periodontitis, we investigated whether GCA neutralization could attenuate disease progression. Ligatured mice received GCA‐NAb (1 mg/kg) via tail vein injection every other day for 2 weeks as reported (Figure S5A) [29, 30, 36]. GCA‐NAb treatment markedly reduced proinflammatory cytokine expression in gingival tissues compared with PBS controls (Figure S5B,C). Flow cytometry further revealed a significant decrease in CD16/32^+^ macrophages in the gingiva (Figure S5D,E). Micro‐CT analysis showed increased residual alveolar bone volume in GCA‐NAb‐treated mice (Figure S5F), accompanied by fewer multinucleated TRAP‐positive osteoclasts (Figure S5G,H). Consistently, H&E and Masson's trichrome staining demonstrated improved periodontal architecture and preserved collagen integrity (Figure S5I,J). Together, these results indicate that GCA‐NAb alleviates periodontal inflammation and bone loss, supporting its therapeutic potential in periodontitis.

### 2.8 Localized Hydrogel Delivery of GCA‐NAb Controls Inflammation and Maintains Periodontal Integrity and Bone Structure

**FIGURE 7:**
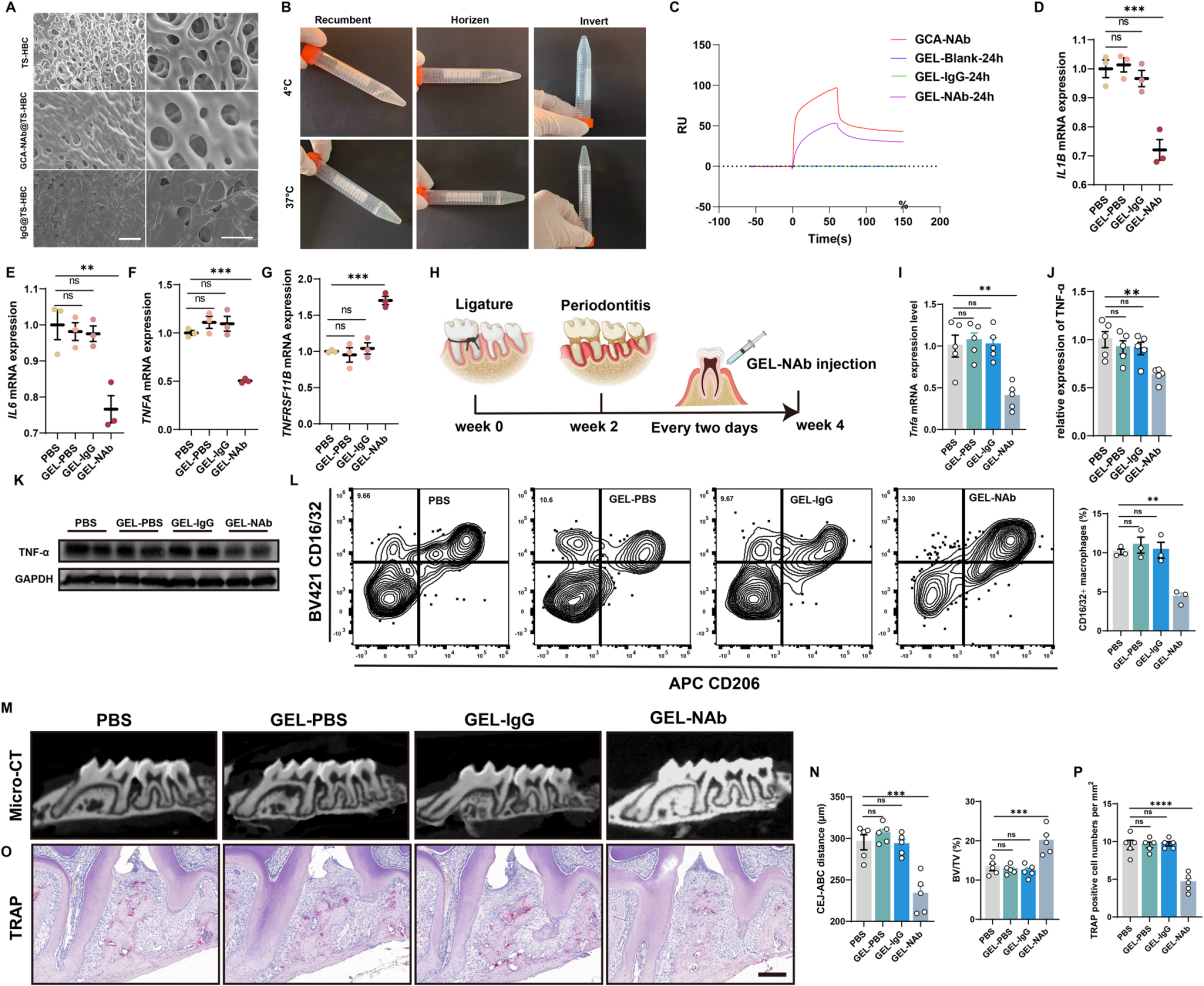
Hydrogel delivery of GCA‐NAb controls inflammation and maintains periodontal integrity and bone structure. (A) Representative scanning electron microscopy (SEM) images of blank TS‐HBC hydrogel, IgG@TS‐HBC (GEL‐IgG), and GCA‐NAb@TS‐HBC hydrogels (GEL‐NAb) (scale bars, 50 µm and 20 µm). (B) Inverted vial assay demonstrating the thermosensitive sol–gel transition behavior of GCA‐NAb@TS‐HBC at increasing temperatures. (C) Surface plasmon resonance (SPR) analysis of the binding affinity between rGCA and free GCA‐neutralizing antibody (GCA‐NAb), as well as hydrogel‐released media from GEL‐Blank, GEL‐IgG, and GEL‐NAb. (D–G) RT‐qPCR analysis of *IL1B, IL6, TNFA*, and *TNFRSF11B* in HGFs preincubated with release media from PBS@TS‐HBC (GEL‐PBS), GEL‐IgG, or GEL‐NAb, followed by stimulation with recombinant GCA (rGCA) (*n* = 3). (H) Schematic illustration of the ligature‐induced periodontitis model and subsequent GEL‐NAb treatment protocol. Ligatures were placed for 2 weeks to induce periodontitis and were then removed, followed by local periodontal injections of PBS, GEL‐PBS, GEL‐IgG, or GEL‐NAb for an additional 2 weeks. Created with BioRender.com. (I) RT‐qPCR analysis showing the mRNA expression of *Tnfa* in gingival tissues (*n* = 5). (J,K) Western blot analysis and quantification of TNF‐α protein in gingival tissues (*n* = 5). (L) Flow cytometric analysis and quantification of CD16/32^+^ proinflammatory macrophages in gingival tissues (*n* = 3). (M,N) Representative micro‐CT images (M) and quantitative analysis (N) of alveolar bone loss, including CEJ–ABC distance and BV/TV (*n* = 5). (O,P) Representative TRAP staining (O) of alveolar bone sections and quantification (P) of TRAP‐positive cells per millimeter of alveolar bone surface (scale bars, 200 µm; *n* = 5). Data are shown as the mean ± SEM. *n* indicates the number of biologically independent samples examined. Statistical significance was determined using one‐way ANOVA followed by Dunnett's test for comparison with the PBS group (D–G,I,J,L,N,P). *p < 0.05, **p < 0.01, ***p < 0.001, ****p < 0.0001.

Building on our finding that neutralization of GCA significantly alleviated periodontal inflammation and bone structural deterioration, we further explored a localized therapeutic strategy. To this end, we engineered the TS‐HBC hydrogel capable of delivering GCA‐NAb (GCA‐NAb@TS‐HBC) directly to periodontal sites. Scanning electron microscopy (SEM) analysis showed that loading with GCA‐NAb altered the porous microstructure of TS‐HBC hydrogels, leading to denser networks and thicker fibers, indicating successful drug incorporation (Figure 7A). The sol–gel transition test showed that GCA‐NAb@TS‐HBC was fluid at 4°C but rapidly gelled within 60 s at 37°C, with reversible temperature responsiveness (Figure 7B). Moreover, the cumulative release profile demonstrated that 0.2 mg/mL GCA‐NAb@TS‐HBC displayed sustained release behavior, reaching a peak at 24 h, confirming its long‐term delivery capacity (Figure S5K). Surface plasmon resonance (SPR) analysis verified the binding affinity of the hydrogel for GCA (Figure 7C). Importantly, fibroblasts were treated with release media collected from GCA‐NAb@TS‐HBC (GEL‐NAb), IgG@TS‐HBC (GEL‐IgG), and PBS@TS‐HBC (GEL‐PBS), followed by stimulation with rGCA. The results showed only the release media from GEL‐NAb effectively neutralized the effects of rGCA, as evidenced by the suppressed induction of proinflammatory cytokines and the concomitant restoration of *TNFRSF11B* expression (Figure 7D–G), indicating the antibody released from GEL‐NAb retains its functional neutralizing activity. Furthermore, biocompatibility assays demonstrated favorable biosafety profiles (Figure S5L,M).

In a mouse ligature‐induced periodontitis model (Figure 7H), local administration of GEL‐NAb significantly reduced gingival inflammation compared to GEL‐IgG, GEL‐PBS, and PBS controls (Figure 7I–K). This anti‐inflammatory effect was evidenced by decreased proinflammatory cytokine expression and a marked reduction in CD16/32^+^ macrophages (Figure 7L). Micro‐CT imaging demonstrated improved alveolar bone parameters relative to PBS controls (Figure 7M,N). Histologically, TRAP staining revealed fewer TRAP‐positive osteoclasts (Figure 7O,P). Consistently, H&E and Masson's trichrome staining demonstrated improved periodontal architecture and preserved collagen integrity (Figure S5N–P). Collectively, localized hydrogel delivery of GCA‐NAb effectively suppresses periodontal inflammation and mitigates bone loss, thereby alleviating disease progression.

## 3 Discussion

Previous studies have shown that senescent myeloid‐derived GCA promotes bone loss by suppressing osteogenic differentiation of bone marrow stromal cells while inhibiting osteoclast formation [29]. Beyond this direct mechanism, our study identifies a fibroblast‐dependent pathway through which GCA disrupts alveolar bone homeostasis. Under physiological conditions, gingival fibroblasts help maintain periodontal tissue integrity and bone homeostasis; however, chronic inflammatory stress drives their transition toward a proinflammatory phenotype that remodels the local immune microenvironment and accelerates periodontal tissue destruction [22, 26, 37, 38]. In this context, we demonstrate that GCA acts on gingival fibroblasts to induce inflammatory reprogramming, amplify NF‐κB‐dependent signaling, and suppress osteoclast‐inhibitory factors. Notably, the resulting fibroblast secretome, particularly enriched in proinflammatory cytokines, potently promotes osteoclastogenesis and drives alveolar bone loss. We therefore propose that GCA‐driven inflammatory crosstalk between myeloid cells and gingival fibroblasts constitutes a key mechanism underlying pathological bone remodeling in periodontitis.

MYH9 is an actin‐binding protein involved in multiple cellular processes and has recently been recognized as a phosphorylation‐dependent transporter that regulates nuclear signaling [35, 39]. Here, we show that activation of the GCA–CD44 axis induces MYH9 phosphorylation, enabling its interaction with the NF‐κB subunit p65 and promoting p65 nuclear translocation and activation, thereby driving proinflammatory gene expression in fibroblasts. Using phospho‐mimetic MYH9 mutants and MYH9 inhibition, we demonstrate that MYH9 phosphorylation is both necessary and sufficient for the GCA‐triggered inflammatory cascade. These findings identify MYH9 as a previously unrecognized mediator of inflammatory fibroblast activation.

Building on GCA as a therapeutic target, we treated periodontitis mice with GCA‐NAb to assess its periodontal therapeutic effects. Our previous studies have shown that this dose is well‐tolerated, causing no weight change over two months while exerting anti‐inflammatory actions in metabolic tissues [30]. In addition, long‐term GCA intervention also did not alter circulating inflammatory cytokines in aged mouse models [29]. Collectively, these findings suggest that systemic administration of GCA‐NAb has a favorable safety profile and does not overtly perturb systemic inflammatory homeostasis under the conditions tested. In this study, we further employed an injectable thermosensitive hydrogel (TS‐HBC) loaded with GCA‐NAb for localized delivery. At 4°C, TS‐HBC remains liquid for easy administration, while at body temperature it rapidly undergoes gelation to ensure stable retention in periodontal pockets and sustained drug release [40]. Studies have shown that similar hydrogel systems can achieve sustained local release of therapeutic agents for at least 24–48 h in vivo [41], with the effects largely confined to the local tissue [42], indicating that the TS‐HBC platform is capable of facilitating efficient and localized protein delivery. While GCA‐NAb@TS‐HBC demonstrates promising therapeutic potential in periodontitis, further investigation is needed to characterize its in vivo release kinetics and local concentration profiles in order to meet precise clinical requirements.

While our study provides important insights into the role of myeloid‐derived GCA in periodontitis, several limitations must be acknowledged. First, our mechanistic investigations focused primarily on gingival fibroblasts as the principal cellular target of GCA, leaving the potential roles of other cell types unexplored. Second, although our findings are derived from a mouse model and a limited number of human tissues, direct evidence linking GCA expression to the clinical onset of periodontitis remains absent. Third, we did not examine whether GCA neutralization influences systemic immune priming beyond the oral cavity. Finally, the exclusive reliance on the ligature‐induced periodontitis model, without exploring alternatives such as infection‐induced or diabetic models, may constrain the broader applicability of our conclusions.

Thus, our study identifies GCA as a critical driver of periodontitis and highlights its potential as a therapeutic target for mitigating inflammatory bone loss and preserving periodontal health.

## 4 Conclusion

1. GCA is markedly elevated in gingival tissues, gingival crevicular fluid, saliva, and serum from periodontitis patients, with its levels positively correlating with key inflammatory cytokines and clinical indicators of disease severity, supporting its potential role as a disease‐associated indicator.
2. Mechanistically, myeloid‐derived GCA activates the CD44–MYH9**–**NF‐κB axis in gingival fibroblasts, driving proinflammatory signaling and promoting osteoclastogenesis, which collectively exacerbate periodontal inflammation and pathological bone remodeling.
3. Localized delivery of a GCA‐neutralizing antibody via an injectable thermosensitive hydrogel effectively attenuates disease progression in vivo. Together, these findings validate GCA as a therapeutically actionable target and highlight the translational potential of hydrogel‐based local delivery of GCA‐NAb for precision treatment of periodontitis.

## 5 Materials and Methods

### 5.1 Human Sample Collection

Gingival tissues and peripheral blood were collected from patients diagnosed with periodontitis (*n* = 20, aged 24–41 years) and from systemically healthy individuals (control group, *n* = 20, aged 19–39 years) at Xiangya Hospital. The study was approved by the Medical Ethics Committee of Xiangya Hospital (approval number: 2025081338), and informed consent was obtained from all participants in accordance with the Declaration of Helsinki guidelines. Inclusion criteria for the periodontitis group were as follows: (1) age 18–65 years; (2) presence of teeth requiring extraction due to periodontitis‐associated mobility (diagnosed according to the 2018 classification and definition of periodontitis [43]); (3) no use of antibiotics, nonsteroidal anti‐inflammatory drugs, or immunosuppressants within the past 3 months; (4) no periodontal treatment within the past 3 months; (5) absence of diabetes, uncontrolled systemic diseases, smoking, or betel nut chewing; (6) no history of bisphosphonate therapy; (7) for female patients, not during menstruation, pregnancy, or lactation. Inclusion criteria for the control group were as follows: (1) age 18–65 years; (2) teeth requiring extraction for reasons unrelated to periodontitis (e.g., impacted teeth or orthodontic treatment); (3) full‐mouth probing depth ≤ 3 mm, no attachment loss due to periodontitis, and bleeding on probing < 20%; (4) absence of diabetes, uncontrolled systemic diseases, smoking, or betel nut chewing; (5) no history of bisphosphonate therapy; (6) for female patients, not during menstruation, pregnancy, or lactation. Gingival tissues obtained during tooth extraction were immediately rinsed with sterile PBS. Portions of the tissues were snap‐frozen in liquid nitrogen for subsequent RNA and protein analyses, while other portions were fixed in 4% paraformaldehyde for histological and immunofluorescence staining. Additional gingival samples were freshly processed for single‐cell RNA sequencing or used for the isolation and culture of primary human gingival fibroblasts. Peripheral blood (3–5 mL) was drawn into serum separation tubes, centrifuged at 3000 × *g* for 10 min after clotting, and the resulting serum was aliquoted and stored at −80°C until use. Additionally, unstimulated whole saliva and GCF were collected from all participants. Saliva samples were clarified by centrifugation and stored at −80°C. GCF was collected from the deepest pocket (periodontitis group) or a healthy site (control group) using sterile paper strips, eluted, and stored at −80°C for subsequent analysis.

### 5.2 Animal

*Gca*‐Cre‐EGFP, *Gca*‐KO, and *Gca* ^flox/flox^ mice were obtained from Cyagen (China). Lyz2‐Cre mice (Stock No. 004781‐B6.129P2‐Lyz2tm1(cre)Ifo/J) were purchased from the Jackson Laboratory (USA). Additionally, 8‐week‐old C57BL/6J mice were obtained from Beijing Vital River Laboratory Animal Technology. All animals were maintained under SPF conditions at 22–24°C with a 12 h light/dark cycle and free access to chow and water. All animal care protocols and experiments were reviewed and approved by the Institutional Animal Care and Use Committee of the Laboratory Animal Research Center of Central South University (approval number: CSU‐2024‐0112). Animal experiments were conducted in accordance with the Animal Research: Reporting of In Vivo Experiments (ARRIVE) guidelines. Group assignment was randomized, and outcome assessments were performed by investigators blinded to the treatment or genotype.

For the generation of gingival fibroblast‐targeted *Cd44* or *Myh9* knockdown mice, adeno‐associated virus (AAV) vectors targeting Cd44 or Myh9, driven by a Col1a1 promoter, were locally administered to gingival tissues. Viral delivery was performed by local gingival injection, aiming to preferentially transduce resident gingival fibroblasts. All AAV vectors were obtained from Hanbio Biotechnology (China). One month after virus injection, mice were subjected to periodontal disease modeling or indicated treatments, followed by gingival tissue collection for downstream molecular and histological analyses.

### 5.3 Ligature‐Induced Periodontitis Model in Mice

To induce experimental periodontitis, a sterile silk suture was ligated around the maxillary first molar. Following gentle elevation of the gingival margin using a micro‐explorer, the ligature was passed interproximally and secured with a double knot on the palatal side to ensure stable subgingival placement without inducing tooth mobility or strangulation of the crown. Control mice underwent identical anesthesia and gingival manipulation but did not receive a ligature. All ligatures were maintained in place for 14 days. Mice were monitored daily for ligature retention, oral hygiene, and any signs of bleeding. Dislodged ligatures were replaced within 24 h. Any mouse that lost its ligature for more than 24 h was excluded from the study based on predefined criteria [44, 45].

### 5.4 Single‐Cell RNA Sequencing

#### 5.4.1 Sample Preparation

Gingival tissues were obtained from two patients with periodontitis and two healthy controls. Fresh tissues were washed with ice‐cold PBS and enzymatically dissociated into single‐cell suspensions using the SeekMate Tissue Dissociation Kit (SeekGene). DNase I treatment was applied when necessary to reduce viscosity. After red blood cell lysis, viable cells were counted using a fluorescence cell counter (AO/PI staining), and suspensions were adjusted to 1×10^6^ cells/mL in PBS with 0.04% bovine serum albumin.

#### 5.4.2 Library Construction and Sequencing

Single‐cell RNA‐seq libraries were prepared using the SeekOne Digital Droplet Single Cell 3′ Library Preparation Kit (SeekGene, K00202). Briefly, single cells were encapsulated with barcoded hydrogel beads in oil droplets, followed by reverse transcription at 42°C for 90 min and inactivation at 85°C for 5 min. After droplet breakage, cDNA was purified, amplified, and subjected to fragmentation, end repair, adaptor ligation, and indexed PCR. Final libraries were cleaned with SPRI beads, quantified with Qubit and Bioanalyzer, and sequenced on an Illumina NovaSeq 6000 platform with PE150 read length.

#### 5.4.3 Single‐Cell RNA‐Seq Data Processing and Analysis

Single‐cell RNA‐seq data were processed and analyzed using a standardized pipeline implemented on the SeekGene cloud analysis platform, following analytical principles consistent with the Seurat framework. Raw sequencing reads were aligned to the human reference genome (GRCh38), and gene–cell count matrices were generated for downstream analyses.

Low‐quality cells were excluded if they expressed fewer than 200 genes, more than 6000 genes, or exhibited mitochondrial gene content exceeding 20%. Genes detected in fewer than three cells were removed. Data were log‐normalized with a scaling factor of 10,000, and the top 2000 highly variable genes were selected for subsequent analysis. Expression values were scaled while regressing out total UMI counts and mitochondrial gene percentage.

To minimize intersample variability, datasets from different individuals were integrated prior to clustering. Principal component analysis was performed on the integrated dataset, and the top 30 principal components were selected for downstream analyses. Cell clustering was conducted using a graph‐based algorithm, and clusters were visualized using Uniform Manifold Approximation and Projection (UMAP).

Cell types were annotated based on established canonical marker genes for immune and stromal populations. Differential gene expression between cell subsets was assessed using the Wilcoxon rank‐sum test with false discovery rate (FDR) correction. Subclustering analyses were performed for macrophages and neutrophils to resolve cellular heterogeneity and identify GCA‐enriched populations.

For gene expression analysis, violin plots were generated to visualize the distribution of GCA expression levels across different groups, and dot plots were used to illustrate GCA expression across annotated cell populations. Additionally, heatmaps of differentially expressed genes were generated for GCA^+^ versus GCA^−^ myeloid cells, as well as macrophage_2 and neutrophil_5 clusters, to identify GCA‐driven transcriptional signatures. To identify the functional pathways most enriched in gingival fibroblasts from periodontitis patients, we performed Hallmark pathway enrichment analysis using differentially expressed genes.

### 5.5 Cell Culture and Treatment

Primary human gingival fibroblasts (HGFs) were isolated from gingival tissues obtained from healthy donors or periodontitis patients during tooth extraction. Briefly, gingival samples were immediately transferred into pre‐chilled medium containing antibiotics. After thorough washing to remove blood and debris, epithelial tissue was carefully trimmed away, and samples were minced into ∼1 mm^3^ fragments. The tissue fragments were digested with 3 mg/mL collagenase type I and 4 mg/mL neutral protease II at 37°C for 40 min. Digestion was terminated by adding complete medium containing 10% fetal bovine serum (FBS), followed by centrifugation at 1000 rpm for 5 min. The resulting tissue pieces were transferred into T25 flasks prewetted with complete DMEM (20% FBS, 1% penicillin–streptomycin) and allowed to adhere for 4 h before being fully immersed in medium. Cultures were maintained at 37°C in 5% CO_2_. Nonadherent tissues were removed after 3 days, and medium was replaced every 3 days. Outgrowth of spindle‐shaped fibroblasts was typically observed within 7–10 days. Human embryonic kidney (HEK) 293T and murine macrophage cell line RAW264.7 were purchased from Procell Life Science & Technology Co., Ltd. (Wuhan, China).

HGF, HEK 293T, and RAW264.7 cells were cultured in DMEM containing 10% FBS, 100 U/mL penicillin, and 100 µg/mL streptomycin at 37°C in a 5% CO_2_ humid atmosphere.

Recombinant GCA protein was purchased from MedChemExpress. According to the manufacturer's specifications, the protein purity was greater than 95% as determined by SDS‐PAGE, and the endotoxin level was less than 1.0 EU/µg. These quality controls ensure that the biological effects observed in this study are attributable to the GCA protein itself rather than contaminating proteins or endotoxin‐induced nonspecific immune activation. HGFs and NCTC Clone 929 Cells were treated with recombinant GCA protein or vehicle control at the indicated concentrations and durations for downstream analyses.

### 5.6 Bone Marrow‐Derived Macrophage Isolation and Osteoclast Differentiation

BMMs were isolated from femurs and tibias of wild‐type mice. Bone marrow cells were flushed with α‐MEM supplemented with 10% FBS and filtered through a 70 µm cell strainer. After red blood cell lysis, cells were seeded in complete α‐MEM containing 10% FBS, 100 U/mL penicillin, 100 µg/mL streptomycin, and recombinant macrophage colony‐stimulating factor (M‐CSF, 30 ng/mL). Nonadherent cells were removed after 24 h, and adherent BMMs were cultured for an additional 2–3 days before osteoclast induction.

For osteoclast differentiation, BMMs were cultured in α‐MEM supplemented with M‐CSF (30 ng/mL) and receptor activator of nuclear factor‐κB ligand (RANKL, 50 ng/mL). Recombinant GCA protein was added where indicated throughout the differentiation period. Culture medium was replaced every 2 days, and osteoclast formation was assessed after 5–7 days by tartrate‐resistant acid phosphatase (TRAP) staining. The area of TRAP‐positive multinucleated cells (MNCs) was quantified using ImageJ software.

### 5.7 HGF‐Conditioned Medium Preparation and Indirect Osteoclastogenesis Assay

HGFs derived from periodontitis patients (PD‐HGFs) were seeded in 6‐well plates and allowed to adhere overnight. For rGCA stimulation experiments, cells were treated with recombinant GCA protein (rGCA) or PBS control in low‐serum DMEM (1% FBS) for 24 h. For Angstrom 6 experiments, PD‐HGFs were pretreated with Angstrom 6 or vehicle control in low‐serum DMEM for 24 h. Conditioned medium was then collected, centrifuged at 1000 × *g* for 10 min to remove cellular debris, and stored at −80°C until use. The detailed procedure was described previously [46].

BMMs were isolated from mice and cultured in osteoclast induction medium containing M‐CSF (30 ng/mL) and RANKL (50 ng/mL). BMMs were supplemented with PD‐HGF conditioned medium or control medium as indicated. Osteoclast differentiation was assessed after 5–7 days by TRAP staining. The area of TRAP‐positive MNCs was quantified using ImageJ software.

### 5.8 TRAP Staining and Quantification

Osteoclast formation was evaluated by TRAP staining using a commercial TRAP staining kit according to the manufacturer's instructions. TRAP‐positive multinucleated cells containing three or more nuclei were identified as mature osteoclasts and the area of TRAP‐positive MNCs was quantified using ImageJ software.

### 5.9 ELISA

The concentrations of inflammatory cytokines were quantified using enzyme‐linked immunosorbent assay (ELISA) kits according to the manufacturer’s protocols. In human biological samples, including serum, saliva, and GCF, levels of IL‐6 and TNF‐α were measured.

In HGF‐conditioned media, levels of IL‐1β, IL‐6, TNF‐α, and OPG were determined to assess fibroblast‐derived inflammatory and osteoclastogenic mediators following GCA treatment.

### 5.10 Receiver Operating Characteristic (ROC) Curve Analysis

To assess the diagnostic performance of circulating GCA for distinguishing periodontitis patients from healthy controls, ROC curve analysis was performed using serum GCA concentrations. ROC curves and the AUC were generated with the rocit package in R (version 4.4.2; R Foundation for Statistical Computing, Vienna, Austria). Sensitivity and specificity at various thresholds were calculated, and the optimal cutoff value was determined using the Youden index (sensitivity + specificity – 1). The 95% confidence interval (CI) of the AUC was calculated using the nonparametric bootstrap method with 1000 iterations. All analyses were conducted in R software.

### 5.11 Plasmid and siRNA Transfection

Plasmids encoding HA‐tagged CD44, Myc‐tagged GCA, His‐tagged p65 and Flag‐tagged MYH9, as well as MYH9 phosphorylation mutants (Ser1943 phospho‐mimetic), together with siRNAs targeting CD44 or MYH9 and corresponding negative controls, were obtained from YouBio Technology Corporation (Shanghai, China).

For transfection of siRNA or plasmids, cells were seeded in 6‐well plates and transfected with Lipofectamine 2000 (Thermo Scientific). Western blot (WB) or RT‐qPCR was used to detect transfection efficiency.

### 5.12 RNA Sequencing

HGFs were treated with recombinant GCA protein or vehicle control for 24 h. Total RNA was extracted using TRIzol reagent according to the manufacturer's instructions. RNA concentration and integrity were assessed using an Agilent Bioanalyzer, and samples meeting quality requirements were used for library preparation.

RNA‐seq libraries were constructed using a poly(A)‐enriched protocol and sequenced on an Illumina platform to generate paired‐end reads. Raw sequencing data were subjected to quality control and mapped to the human reference genome. Gene expression levels were quantified, and downstream analyses, including differential gene expression analysis and pathway enrichment analysis, were performed using standard bioinformatics pipelines.

### 5.13 16S rRNA Gene Sequencing of Oral Microbiota

Oral microbiota analysis was performed in ligatured wild‐type and GCA‐deficient mice using 16S rRNA gene sequencing. Oral samples were collected under sterile conditions, and total microbial DNA was extracted using a commercial bacterial DNA extraction kit according to the manufacturer's protocol.

The V3‐V4 regions of the bacterial 16S rRNA gene were amplified and sequenced on an Illumina platform. Raw sequencing data were processed for quality filtering, sequence clustering, and taxonomic assignment using standard analysis workflows. Microbial diversity metrics and taxonomic composition were analyzed for downstream comparison.

### 5.14 Mass Spectrometry Analysis

Mass spectrometry analysis was performed by in‐gel digestion followed by liquid chromatography‐tandem mass spectrometry (LC‐MS/MS). Briefly, protein bands were excised, destained, washed, dehydrated with acetonitrile, reduced with DTT, and alkylated with iodoacetamide. After overnight digestion with sequencing‐grade trypsin (37°C), peptides were extracted, desalted using C18 columns, lyophilized, and reconstituted in 0.1% formic acid. Peptide mixtures were separated on an EASY‐nLC 1000 UPLC system with a C18 column (1.8 µm, 0.15×100 mm) using a 98‐min gradient (0.1% formic acid in water, solvent A; 100% acetonitrile, solvent B) at a flow rate of 600 nL/min, and analyzed on a Q Exactive HF‐X mass spectrometer (Thermo Fisher Scientific). Full MS scans (m/z 350–1600, resolution 30,000) were followed by data‐dependent HCD MS/MS scans of the top 20 precursors (resolution 17,500, NCE 35). Raw data were processed using Proteome Discoverer 3.1 with searches against the UniProt Homo sapiens database, trypsin specificity (two missed cleavages), carbamidomethylation (C, fixed), oxidation (M, variable), precursor tolerance 20 ppm, and fragment tolerance 0.05 Da. High‐confidence peptides were filtered using a *q*‐value‐based FDR threshold.

### 5.15 Molecular Docking

The crystal structure of GCA (PDB ID: 1K94) was obtained from the Protein Data Bank, while the predicted structure of CD44 was generated using AlphaFold. Protein preparation for docking was carried out with AutoDockTools 1.5.7, during which water molecules were removed and polar hydrogens were added. Protein‐protein docking was performed using the GRAMM web server, and the resulting complexes were further refined with AutoDockTools by eliminating water molecules and adding polar hydrogens. Protein‐protein interactions were visualized in PyMOL.

### 5.16 Micro‐CT Analysis

Mouse maxilla tissues were fixed in 4% paraformaldehyde at room temperature for 24 h and scanned on a Quantum GX2 micro‐CT system (PerkinElmer). Scanning parameters were set to 90 kV, 80 µA for 4 min with a field of view of 18 mm. Projection data were reconstructed into 3D volumes using the instrument's reconstruction software.

For quantitative analysis of alveolar bone loss, the distance between the cementoenamel junction (CEJ) and the alveolar bone crest (ABC) was measured as a standardized indicator of periodontal bone destruction. Measurements were performed on the buccal and palatal aspects of the maxillary molars. The CEJ was defined as the anatomical junction between enamel and cementum, and the ABC was defined as the most coronal point of the alveolar bone surrounding each tooth.

CEJ–ABC distances were measured at six predetermined sites per mouse (mesial and distal aspects of the first molar), and the mean value was calculated for each specimen. All measurements were conducted using blinded analysis to minimize observer bias.

In addition, bone volume to total volume (BV/TV) was calculated to assess changes in alveolar bone microarchitecture. A standardized region of interest surrounding the maxillary molar alveolar bone was defined, and BV/TV was quantified using the micro‐CT analysis software according to the manufacturer's instructions.

### 5.17 Flow Cytometry

The cell suspension was prepared and used for fluorescence‐activated cell sorting (FACS) as described previously [47]. Briefly, gingival tissues were minced and digested in RPMI‐1640 containing 1.5 mg/mL collagenase II and 0.2 mg/mL DNase I for 30 min at 37°C with gentle agitation. After filtering through a 70 µm strainer, centrifugation, and erythrocyte lysis, single‐cell suspensions were obtained and resuspended in PBS containing 2% FBS. For the assessment of Gca expression, gingival cells isolated from *Gca*‐Cre‐EGFP reporter mice were directly analyzed for GFP fluorescence using a DxP Athena flow cytometer. For macrophage phenotyping in ligature‐induced periodontitis mice, single‐cell suspensions were incubated with Brilliant Violet (BV) 510‐ Zombie (423101, BioLegend) for 15 min at room temperature. After washing with PBS, single‐cell suspensions were stained with APC‐Cy7‐CD45 (103116, BioLegend), PE‐Cy7‐F4/80 (123114, BioLegend), FITC‐CD11b (101206, BioLegend), BV421‐CD16/32 (101332, BioLegend), and APC‐CD206 (141708, BioLegend) antibodies for 20–30 min at 4°C in the dark. After two washes, cells were resuspended in stain buffer and analyzed on the DxP Athena system. Data were processed with FlowJo software (v10).

### 5.18 Isolation and Adoptive Transfer of GCA^+^ and GCA^−^ Myeloid Cells

Bone marrow cells were harvested from ligatured *Gca*‐Cre‐EGFP mice under sterile conditions. Single‐cell suspensions were prepared and subjected to FACS. GCA^+^ myeloid cells (CD11b^+^Ly6G^−^Ly6C^+^EGFP^+^) and GCA^−^ myeloid cells (CD11b^+^Ly6G^−^Ly6C^+^EGFP^−^) were sorted using a FACS Aria cell sorter (BD Biosciences). A total of 2×10^6^ sorted cells were intravenously injected into recipient mice subjected to experimental periodontitis.

### 5.19 Histological and Immunohistochemical Analysis

Human gingival tissues were fixed in 4% paraformaldehyde for 24–48 h. Mouse maxillary samples (containing bone and teeth) were fixed in 4% paraformaldehyde for 48 h and then decalcified in 15% EDTA (pH 7.4), with the solution changed every 2 days. Decalcification was continued for approximately 1–3 weeks, until complete demineralization was confirmed by the absence of resistance upon needle puncture. After decalcification and dehydration, paraffin embedding, and sectioning at 5 µm, H&E staining was performed to assess general morphology. Collagen deposition was evaluated by Masson's trichrome staining (Solarbio), and osteoclast activity was assessed by TRAP staining (Solarbio), following the manufacturer's protocols. For immunohistochemical (IHC) analysis, paraffin‐embedded sections were dewaxed, rehydrated, and subjected to antigen retrieval in citrate buffer. Subsequently, endogenous peroxidases were quenched with 3% H_2_O_2_ and nonspecific binding sites were blocked with 10% BSA. The sections were then incubated overnight at 4°C with primary antibodies against GCA (Abcam, ab231632, 1:200). After washing, the sections were incubated with appropriate secondary antibodies. Histopathological quantification was performed using ImageJ. For IHC analysis, the DAB signal was extracted using the Color Deconvolution plugin, and a fixed threshold was applied to identify positive staining. The percentage of positively stained area was calculated as: %area = (DAB‐positive area / total tissue area) × 100%. Similarly, the collagen volume fraction, represented by the blue‐stained area in Masson's trichrome‐stained sections, was quantified. Additionally, the number of TRAP‐positive multinucleated osteoclasts was counted, and the density was expressed as the number of TRAP‐positive cells per mm^2^ of tissue area.

### 5.20 Immunofluorescence Staining

Maxilla slices and cells were incubated with the primary antibody working solution (diluted with blocking solution) at 4°C for 24 h. The primary antibodies used in this study were as follows: GCA (Abcam, ab231632, 1:200), Pan‐Keratin (Proteintech, 26411‐1‐AP, 1:200), FSP‐1 (Proteintech, 16105‐1‐AP, 1:200), Vimentin (Huabio, ET1610‐39, 1:300), F4/80 (Abcam, ab6640, 1:200), p‐p65 (Cell Signaling Technology, CST8242S,1:200), and MYH9 (Proteintech, 11128‐1‐AP, 1:200). Subsequently, the working solution of the secondary antibody (diluted with blocking solution; 1:200, Thermo Fisher, including Alexa Fluor 488, Alexa Fluor 555) was incubated at room temperature for 1 h. The slides were mounted with antifade mounting medium containing DAPI (Solarbio). Images were visualized on a fluorescence microscope.

### 5.21 GCA Neutralizing Antibody

The GCA neutralizing antibody (SinoBiological, 22ZN27C, 1 mg kg^−1^) was used as previously described [29]. Ligatured mice received GCA‐NAb (1 mg/kg) via tail vein injection every other day for 2 weeks.

### 5.22 RT‐qPCR Analysis

Total RNA was extracted from gingival tissues, bone marrow cells or cultured cells using AG RNAex Pro Reagent (Accurate Biotechnology, Hunan, China), following the manufacturer's instructions. Quantitative real‐time PCR was performed with SYBR Green PCR Master Mix (PE Applied Biosystems) on an Applied Biosystems 7300 system. Primer sequences are listed in Table S2.

### 5.23 Western Blot Analysis

Proteins were extracted from gingival tissues, bone marrow cells, and cultured cells using RIPA lysis buffer (Biosharp) supplemented with protease and phosphatase inhibitors. Protein concentrations were determined by BCA assay (Beyotime). Equal amounts of protein (20–40 µg) were separated by SDS‐PAGE and transferred onto PVDF membranes (Millipore). Membranes were blocked with 5% nonfat milk in TBST for 1 h at room temperature and incubated overnight at 4°C with the following primary antibodies: GCA (Abcam, ab231632, 1:1000), IL‐1β (Santa Cruz, sc‐7884, 1:500), IL‐6 (Santa Cruz, sc‐57315, 1:500), TNF‐α (Santa Cruz, sc‐52746, 1:500), NF‐κB p65 (CST, 8242S, 1:1000), phospho‐NF‐κB p65 (CST, 3033S, 1:1000), MYH9 (Proteintech, 11128‐1‐AP, 1:1000), phospho‐MYH9 (CST, 5026S, 1:1000), Lamin B1 (Proteintech, 12987‐1‐AP, 1:10,000), Flag (Proteintech, 20543‐1‐AP,1:10,000), Myc (Proteintech, 60003‐2‐Ig, 1:10,000), HA (Proteintech, 51064‐2‐AP, 1:10,000), His (Proteintech, 66005‐1‐Ig, 1:10,000), β‐Actin (Proteintech, 66009‐1‐Ig, 1:20,000), and GAPDH (Proteintech, 10494‐1‐AP, 1:10,000). After washing, membranes were incubated with secondary antibodies (Proteintech) for 1 h at room temperature. Protein bands were visualized using the ECL Substrate (Bio‐Rad) and quantified by densitometry using ImageJ.

### 5.24 Preparation and Characterization of GCA‐NAb@TS‐HBC Hydrogels

The GCA‐NAb@TS‐HBC hydrogel was prepared by first dissolving GCA‐NAb powder in PBS under sterile conditions, followed by filtration through a 0.22 µm membrane to obtain solutions at concentrations of 0.1, 0.2, and 0.3 mg/mL. Prechilled TS‐HBC (stored at 4°C, Huizhong International Medical Equipment Co., Ltd.) was then mixed with the GCA‐NAb solutions at a 5:1 (v/v) ratio, gently pipetted ten times to ensure homogeneity, and transferred to a sterile 10 mL centrifuge tube. The mixture was incubated at 37°C in a shaking incubator under two phases: (1) 0–2 h at 100 rpm with intermittent oscillation (5 min on/off) to promote drug diffusion, and (2) 2–24 h at 150 rpm under continuous shaking to complete drug encapsulation. Finally, the sample was allowed to stand at 37°C for 10 min to form the stable GCA‐NAb@TS‐HBC hydrogel. The morphological characteristics of GCA‐NAb@TS‐HBC hydrogel were examined by SEM (Carl Zeiss), while its thermosensitive properties were evaluated by injecting the prechilled (4°C) sol–gel into test tubes and observing reversible gelation through alternating incubation in 37°C and 4°C water baths. For drug release analysis, hydrogels were incubated in nuclease‐free water at 37°C, and supernatant samples were collected at 0, 2, 4, 6, and 8 days. The total protein content in the collected supernatants was quantified using the BCA assay.

For in vitro release analysis, hydrogels were immersed in nuclease‐free water at 37°C, and supernatants were collected at 0, 30 min, 1 h, 2 h, 4 h, 6 h, 12 h, 24 h, 48 h, and 72 h. To evaluate the bioactivity and binding functionality of the released neutralizing antibody, SPR analysis was performed. Briefly, recombinant GCA protein was immobilized on the sensor chip, and binding responses were measured for (i) nuclease‐free water control, (ii) extracts from blank TS‐HBC hydrogel, (iii) extracts from GCA‐NAb@TS‐HBC hydrogel, and (iv) free GCA neutralizing antibody. This approach allowed direct assessment of the specific interaction between released GCA‐NAb and its target protein, thereby confirming the preservation of antibody binding activity following hydrogel encapsulation and release.

### 5.25 Biocompatibility Assessment and Preparation of Hydrogel Extracts

To evaluate cytocompatibility, hydrogel extracts were prepared by incubating GCA‐NAb@TS‐HBC hydrogel (0.2 mg/mL) with complete culture medium at a ratio of 1:10 (w/v) at 37°C for 24 h. The mixture was centrifuged at 1000 × *g* for 10 min, and the supernatant was collected and filtered through a 0.22 µm membrane. The extract was used directly or diluted to the indicated concentrations.

RAW264.7 macrophages were seeded into 96‐well plates at a density of 3×10^3^ cells per well and allowed to adhere for 24 h, followed by treatment with serial dilutions (0%, 12.5%, 25%, 50%, and 100%) of hydrogel extracts for an additional 24 h. Cell viability was assessed using the CCK‐8 assay, and cytocompatibility was further confirmed byLive/Dead cell staining.

### 5.26 Cell Proliferation Assay (CCK‐8)

For recombinant GCA treatment, HGFs were seeded into 96‐well plates at a density of 5.0×10^3^ cells per well and treated with recombinant GCA (0–100 nm) for 24 h. For hydrogel extract treatment, RAW264.7 cells were exposed to different proportions of GCA‐NAb@TS‐HBC hydrogel extracts as described above. CCK‐8 reagent was added for 1 h, and absorbance was measured at 450 nm. Cell viability was calculated relative to untreated controls. All experiments were independently repeated at least three times.

### 5.27 Local Administration of GCA‐NAb@TS‐HBC Hydrogel

For in vivo periodontal application, GCA‐NAb@TS‐HBC hydrogel was freshly prepared and maintained at 4°C in the sol state prior to administration. Based on preliminary in vitro release and biocompatibility evaluations, a final GCA‐NAb concentration of 0.2 mg/mL was selected for subsequent animal experiments.

Under general anesthesia, a small volume (approximately 5–10 µL per injection) of prechilled hydrogel sol was locally injected into the gingival sulcus adjacent to the maxillary molars using a microsyringe. Upon exposure to body temperature, the sol rapidly transitioned into a stable in situ gel. Local administration was performed every 2 days throughout the experimental period.

### 5.28 Statistical Analysis

Data were presented as mean ± standard error of the mean (SEM). Statistical differences between two groups were assessed using an unpaired, two‐tailed Student's *t*‐test. Comparisons among multiple groups were analyzed by one‐way analysis of variance (ANOVA) followed by either Tukey's honestly significant difference test (for all pairwise comparisons) or Dunnett's test (for comparisons against a single control group), as appropriate, or two‐way ANOVA with Tukey's multiple‐comparison test. Spearman's rank correlation coefficient was used to analyze clinical data. Significance levels in all figures are denoted as follows: *p < 0.05; **p < 0.01; ***p < 0.001; ****p < 0.0001. Statistical analysis was performed in GraphPad Prism 9 and R version 4.4.2.

## Author Contributions

M.Z., Y.X., and Z.Y.X. contribute equally to this work. Y.L. and P.Y. designed the projects and supervised this study. M.Z. and Z.Y.X. performed most of the experiments and analyzed the data. Y.X., C.Y.Y., and J.L. participated in the experimental work and data analysis. M.Z. and Y.L. drafted the manuscript. M.Z., Y.L., and P.Y. edited the manuscript. All authors have read and approved the article.

## Funding

This work was supported by the Natural Science Foundation of Hunan Province, China (Grant No. 2022JJ30980).

## Conflicts of Interest

The authors declare no conflicts of interest.

## Supporting information




**Supporting File 1**: advs74897‐sup‐0001‐SuppMat.pdf.

**Supporting File 2**: advs74897‐sup‐0002‐Data.zip.

## Data Availability

The data that support the findings of this study are available from the corresponding author upon reasonable request.
