# Supplementary material for: Myeloid‐Derived Grancalcin Promotes Periodontal Inflammation and Pathological Bone Remodeling in Periodontitis
Source: Adv Sci (Weinh). 2026 Mar 25;13(31):e19267. doi: 10.1002/advs.202519267 (PMC13588070; doi:10.1002/advs.202519267)
Supplement: Supplementary file 1 [file ADVS-13-e19267-s002.pdf]

SUPPLEMENTAL MATERIAL FOR

Myeloid-derived Grancalcin Promotes Periodontal Inflammation and Pathological Bone Remodeling in Periodontitis

Min Zhou[1#], Yao Xiao[1#], Ziyue Xu[2#], Chengyu Yang[2], Jiang Li[2], Ya Liu[1], Ping Yin[2]**

# Min Zhou, Yao Xiao and Ziyue Xu contributed equally to this work

* Corresponding author:

Ping Yin (403668@csu.edu.cn)

Ya Liu (jupiya1109@126.com)

This PDF file includes:

Figure S1. Additional Evidence for GCA Upregulation in Periodontitis.

Figure S2. Supporting Characterization of Gca-KO Mice and Gingival Microbiota Analysis.

Figure S3. Validation of Myeloid-Specific Gca Deletion and Gingival GCA Expression in Periodontitis.

Figure S4. Additional Evidence Supporting rGCA-Mediated Periodontal Inflammation and Bone Remodeling.

Figure S5. Systemic Neutralization of GCA Attenuates Inflammation and Preserves Periodontal Tissues and Bone.

Table S1. Key anitbodies table

Table S2. Nucleotide sequences of primers used for quantitative RT-PCR detection.

Figure S1. Additional Evidence for GCA Upregulation in Periodontitis. (A) Relative GCA mRNA expression in gingival tissues of controls (CON) and periodontitis patients (PD) (n = 5). (B) Representative immunohistochemistry staining and quantitative analysis of GCA in gingiva from CON and PD individuals (scale bar, 100 μm; n = 5). (C) GCA concentration in the saliva from CON and PD individuals (n = 20). (D-E) Correlation analysis between GCA and interleukin-6 (IL-6, D) or tumor necrosis factor-α (TNF-α, E) in saliva from human participants (n = 40). (F-G) Correlation analysis between saliva GCA and probing depth (F) or clinical attachment loss (CAL, G) from PD participants (n = 20). (H) Receiver operating characteristic (ROC) curve analysis of circulating GCA for distinguishing periodontitis patients from healthy controls (n = 40; 20 CON vs 20 PD). The area under the curve (AUC) was 0.731. The optimal cut-off value was 5.530 ng/mL, yielding a sensitivity of 90% and a specificity of 60%. The diagonal dashed line indicates no discriminatory performance (AUC = 0.5). (I) Representative micro-CT–based 3D reconstruction of the maxilla to validate the bone loss of the ligature-induced periodontitis model. (J) Relative Gca mRNA expression in gingival tissues of control (Con) and periodontitis (Pd) mice (n = 5). (K) Representative immunohistochemistry staining (upper) and quantitative analysis (lower) of GCA in gingiva from Con and Pd mice (scale bar, 100 μm; n = 5). (L) Schematic diagram of the breeding strategy for generating Gca-Cre-EGFP mice. (M) Representative genotyping results of Gca-Cre-EGFP mice. Data are presented as mean ± SEM. n indicates the number of biologically independent samples examined. Statistical significance was determined by two-sided Student's t test (A-C, J, K), or two-sided Spearman's correlation analysis (D-G). *$p < 0.05$, **$p < 0.01$, ***$p < 0.001$, ****$p < 0.0001$.

Figure S1

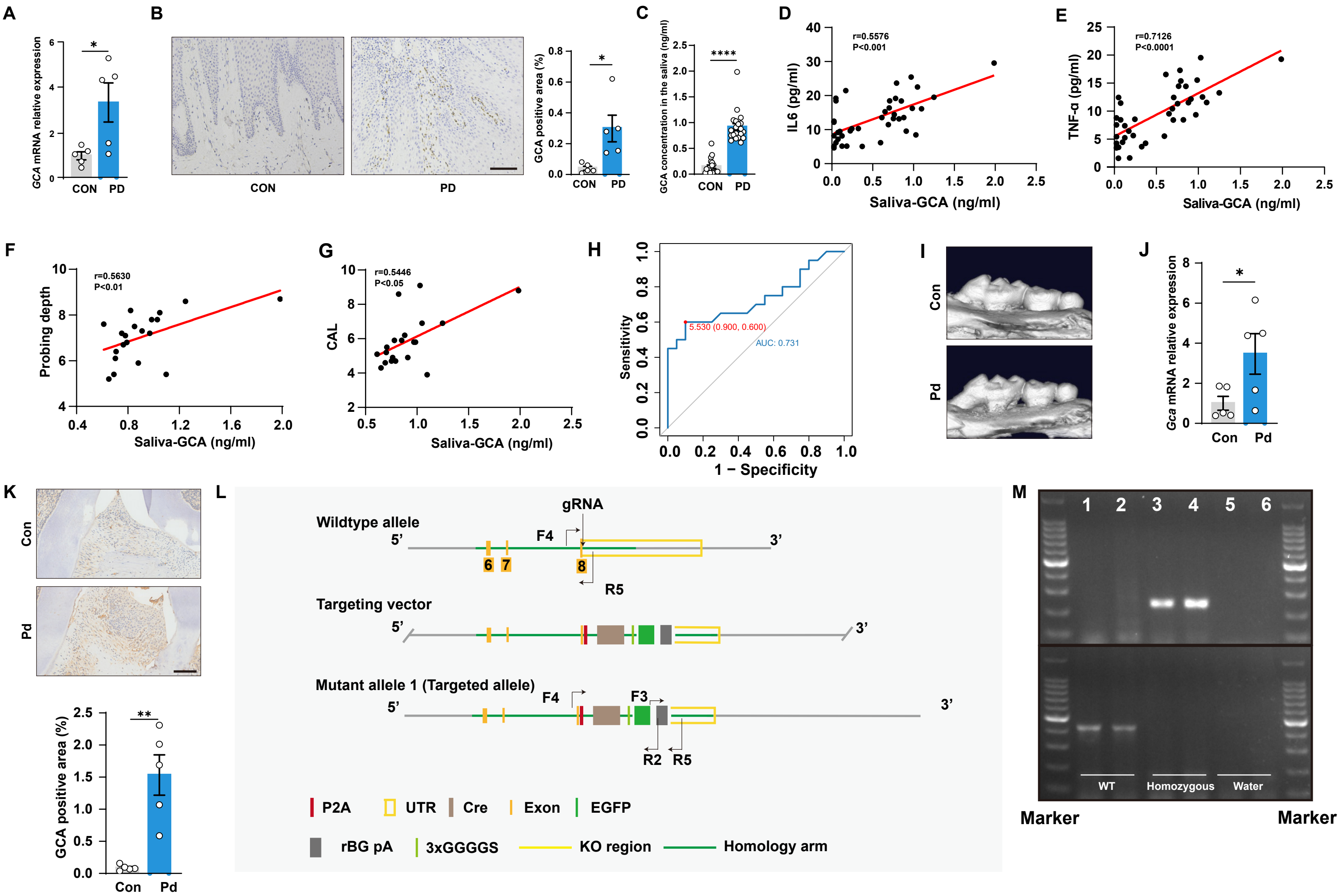

A
GCA mRNA relative expression
CON
PD
B
CON
PD
GCA positive area (%)
C
GCA concentration in the saliva (ng/ml)
D
r=0.5576
P<0.001
IL6 (pg/ml)
Saliva-GCA (ng/ml)
E
r=0.7126
P<0.0001
TNF-α (pg/ml)
Saliva-GCA (ng/ml)
F
r=0.5630
P<0.01
Probing depth
Saliva-GCA (ng/ml)
G
r=0.5446
P<0.05
CAL
Saliva-GCA (ng/ml)
H
Sensitivity
1 − Specificity
5.530 (0.900, 0.600)
AUC: 0.731
I
Con
Pd
J
Gca mRNA relative expression
Con
Pd
K
Con
Pd
GCA positive area (%)
L
gRNA
Wildtype allele
F4
R5
Targeting vector
Mutant allele 1 (Targeted allele)
F3
R2
P2A
UTR
Cre
Exon
EGFP
rBG pA
3xGGGGS
KO region
Homology arm
M
WT
Homozygous
Water
Marker
Marker

Figure S2

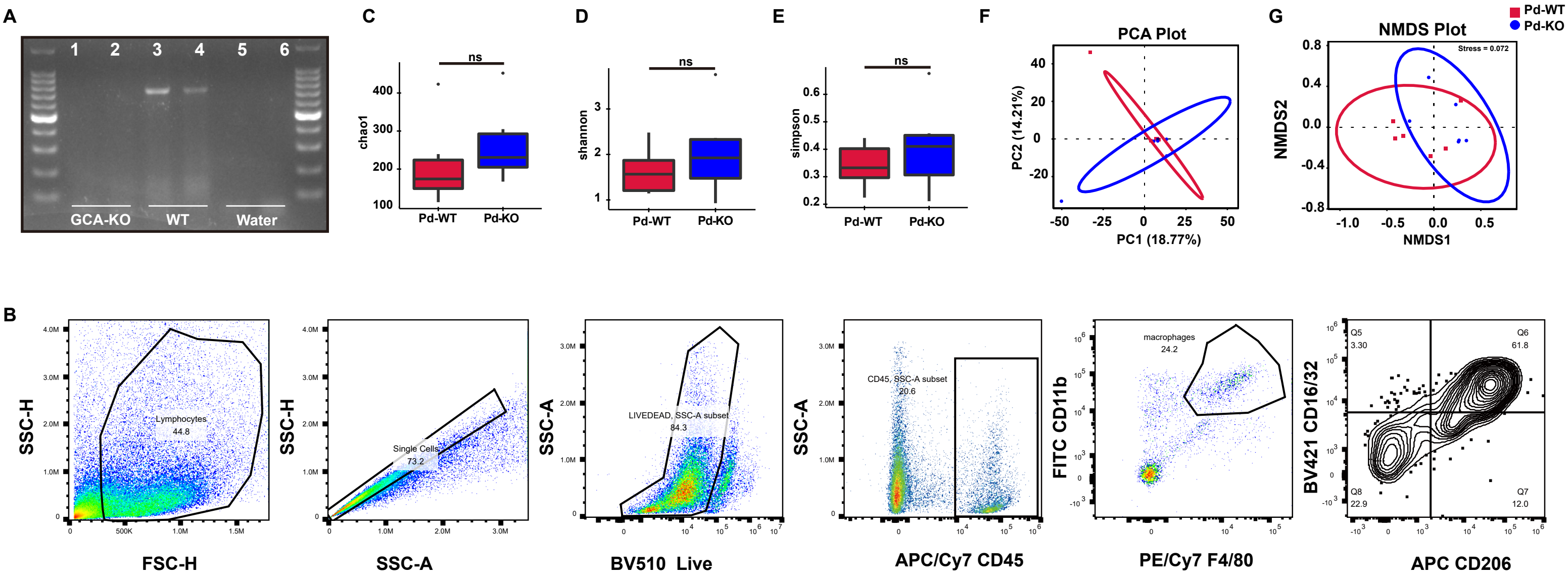

A
1 2 3 4 5 6
GCA-KO
WT
Water
C
ns
chao1
Pd-WT
Pd-KO
D
ns
shannon
Pd-WT
Pd-KO
E
ns
simpson
Pd-WT
Pd-KO
F
PCA Plot
PC2 (14.21%)
PC1 (18.77%)
G
NMDS Plot
Pd-WT
Pd-KO
Stress = 0.072
NMDS2
NMDS1
B
SSC-H
FSC-H
Lymphocytes
44.8
SSC-H
SSC-A
Single Cells
73.2
SSC-A
BV510 Live
LIVEDEAD, SSC-A subset
84.3
SSC-A
APC/Cy7 CD45
CD45, SSC-A subset
20.6
FITC CD11b
PE/Cy7 F4/80
macrophages
24.2
BV421 CD16/32
APC CD206
Q5
3.30
Q6
61.8
Q8
22.9
Q7
12.0

Figure S2. Supporting Characterization of Gca-KO Mice and Gingival Microbiota Analysis. (A) Genotyping results confirming Gca knockout in mice. (B) Flow cytometry gating strategy for identifying CD16/32+ pro-inflammatory macrophages in gingival tissues from ligatured mice. (C-E) Alpha diversity of gingival microbial communities assessed by the Chao1 richness index (C), Shannon diversity index (D), and Simpson diversity index (E) ($n = 6$). (F) Principal component analysis (PCA) based on overall microbial community composition in gingival samples ($n = 6$). (G) Non-metric multidimensional scaling (NMDS) analysis illustrating differences in microbial community structure between ligatured WT and Gca-KO mice ($n = 6$).

Figure S3

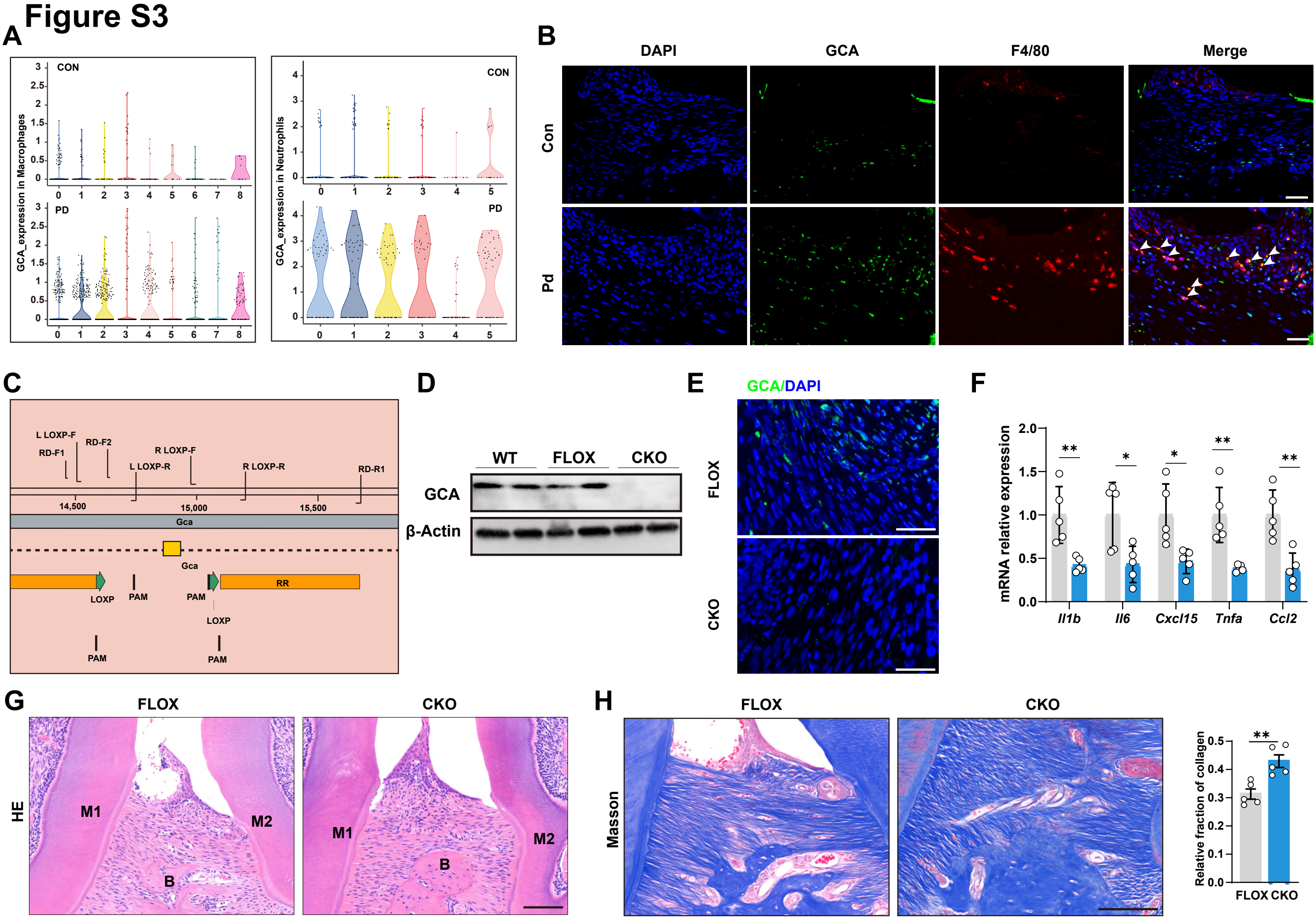
A
CON
PD
GCA_expression in Macrophages
GCA_expression in Neutrophils
B
DAPI
GCA
F4/80
Merge
Con
Pd
C
L LOXP-F
RD-F1
RD-F2
L LOXP-R
R LOXP-F
R LOXP-R
RD-R1
14,500
15,000
15,500
Gca
LOXP
PAM
RR
D
WT
FLOX
CKO
GCA
β-Actin
E
GCA/DAPI
FLOX
CKO
F
mRNA relative expression
Il1b
Il6
Cxcl15
Tnfa
Ccl2
G
FLOX
CKO
HE
M1
M2
B
H
Masson
Relative fraction of collagen
FLOX CKO

Figure S3. Validation of Myeloid-Specific Gca Deletion and Gingival GCA Expression in Periodontitis. (A) Violin plots of GCA expression across macrophage and neutrophil clusters in gingival scRNA-seq data. (B) Immunofluorescence staining of GCA (green) and F4/80 (red) in gingival tissues from control (Con) and periodontitis (Pd) mice, with DAPI nuclear staining (blue) (scale bar, 40 μm). (C) Breeding strategy of $Gca^{flox/flox}$ （FLOX）mice. (D) Representative GCA expression in bone marrow of ligatured FLOX and $Lyz2^{cre}$; $Gca^{flox/flox}$ (CKO) mice as assessed by western blotting. (E) Immunofluorescence staining of GCA in the gingiva of FLOX and CKO ligature-induced periodontitis mice (scale bar, 40 μm). (F) Relative mRNA expression of *Il1b, Il6, Cxcl15, Tnfa and Ccl2* in gingival tissues (n = 5). (G) Representative H&E staining of periodontal tissues from ligatured FLOX and CKO mice (scale bar, 100 μm). M1, the first upper molars; M2, the second upper molars; B, interdental alveolar bone. (H) Representative Masson staining and quantification of collagen volume fraction in gingival tissues based on Masson staining (scale bar, 100 μm; n = 5). Data are presented as mean ± SEM. n indicates the number of biologically independent samples examined. Statistical significance was determined by two-sided Student's t test (F, H). $*p < 0.05$, $**p < 0.01$.

Figure S4

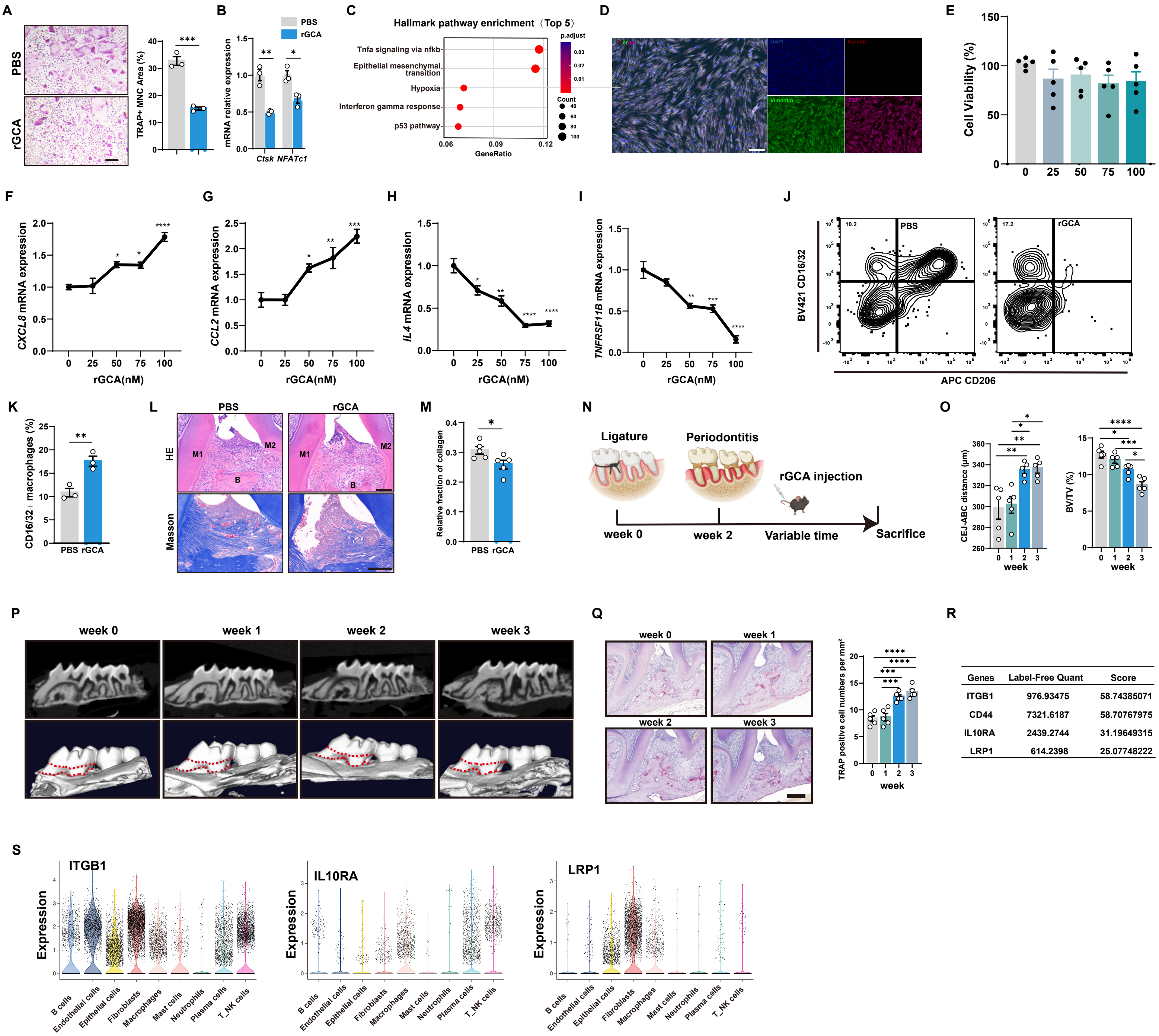
A
PBS
rGCA
TRAP+ MNC Area (%)
B
PBS
rGCA
mRNA relative expression
Ctsk
NFATc1
C
Hallmark pathway enrichment（Top 5）
Tnfa signaling via nfkb
Epithelial mesenchymal transition
Hypoxia
Interferon gamma response
p53 pathway
GeneRatio
p.adjust
Count
D
E
Cell Viability (%)
F
CXCL8 mRNA expression
rGCA(nM)
G
CCL2 mRNA expression
rGCA(nM)
H
IL4 mRNA expression
rGCA(nM)
I
TNFRSF11B mRNA expression
rGCA(nM)
J
PBS
rGCA
BV421 CD16/32
APC CD206
K
CD16/32+ macrophages (%)
PBS rGCA
L
PBS
rGCA
HE
Masson
M1
M2
B
M
Relative fraction of collagen
PBS rGCA
N
Ligature
Periodontitis
rGCA injection
week 0
week 2
Variable time
Sacrifice
O
CEJ-ABC distance (μm)
BV/TV (%)
week
P
week 0
week 1
week 2
week 3
Q
week 0
week 1
week 2
week 3
TRAP positive cell numbers per mm²
week
R
Genes | Label-Free Quant | Score
ITGB1 | 976.93475 | 58.74385071
CD44 | 7321.6187 | 58.70767975
IL10RA | 2439.2744 | 31.19649315
LRP1 | 614.2398 | 25.07748222
S
ITGB1
IL10RA
LRP1
Expression
B cells
Endothelial cells
Epithelial cells
Fibroblasts
Macrophages
Mast cells
Neutrophils
Plasma cells
T_NK cells

Figure S4. Additional Evidence Supporting rGCA-Mediated Periodontal Inflammation and Bone Remodeling. (A) Representative TRAP staining images (left) and quantification (right) of osteoclast formation in bone marrow-derived macrophage (BMM) cultures induced with macrophage colony-stimulating factor (M-CSF) and receptor activator of nuclear factor κB ligand (RANKL), in the presence of PBS or recombinant grancalcin (rGCA). Osteoclastogenesis was quantified as the area (%) of TRAP-positive multinucleated cells (MNCs) (scale bars, 200 μm; n = 3). (B) qPCR quantification of osteoclast-associated genes (*Ctsk* and *NFATc1*) in BMM cultures treated with PBS or rGCA. (C) Bubble plot showing the top five enriched Hallmark pathways based on differentially expressed genes in gingival fibroblasts from healthy controls and periodontitis patients, as determined by single-cell RNA sequencing analysis. Dot size indicates the number of enriched genes, and color represents the significance level of enrichment. (D) Immunofluorescence staining of primary human gingival fibroblasts (HGFs), showing positivity for Vimentin (green) and fibroblast-specific protein (FSP, pink), but negativity for Keratin (red), confirming a mesenchymal rather than epithelial origin. Nuclei were counterstained with DAPI (blue) (scale bars, 200 μm). (E) Cell viability of HGFs treated with different concentrations of recombinant GCA protein, assessed by Cell Count Kit-8 (CCK-8) assay after 24 h, expressed relative to control (0 nM) (n = 5). (F-I) RT-qPCR analysis of *CXCL8*, *CCL2, IL4 and TNFRSF11B* in HGFs treated with rGCA at indicated concentrations (n = 3). (J-K) Flow cytometric analysis (J) and quantification (K) of CD16/32+ pro-inflammatory macrophages in gingival tissues from ligature-induced periodontitis mice treated with PBS or rGCA (250 nM) (n = 3). (L) Representative H&E staining (upper) and Masson staining (lower) of periodontal tissues from ligature-induced periodontitis mice treated with PBS or rGCA (scale bar, 100 μm). M1, the first upper molars; M2, the second upper molars; B, interdental alveolar bone. (M) The quantification of collagen volume fraction in gingival tissues based on Masson staining (n = 5). (N) Schematic illustration of the experimental design. Periodontitis was induced by ligature placement for 2 weeks. After ligature removal,

mice received tail vein injections of recombinant GCA (rGCA; 250 nM in 100 μl PBS) every other day for an additional 0, 1, 2, or 3 weeks to assess the time-dependent effects of rGCA during the post-ligature phase. Created with BioRender.com. (O-P) Representative micro-CT images and three-dimensional reconstructions of maxillae from ligature-induced mice treated with rGCA for the indicated durations, with quantification of the cement-to-enamel junction to alveolar bone crest (CEJ-ABC) distance and bone volume to total volume (BV/TV) ratio (n = 5). (Q) Representative TRAP staining of alveolar bone sections (left) and quantification (right) of TRAP-positive cells per millimeter of alveolar bone surface (scale bars, 200 μm; n = 5). (R) List of four membrane receptor candidates identified by Co-immunoprecipitation combined with mass spectrometry (Co-IP/MS) analysis of membrane proteins from rGCA-treated HGFs. (S) Violin plots showing the expression of three candidate membrane receptor genes (*ITGB1*, *IL10RA* and *LRP1*) across gingival cell types in single-cell RNA-seq data from healthy controls and periodontitis patients. Data are presented as mean ± SEM. n indicates the number of biologically independent samples examined. Statistical significance was determined using two-sided Student's t test (A-B, K, M), one-way ANOVA followed by Dunnett's test for comparison with the control group (0 nM) (E-I) or Tukey's multiple-comparison test (O, Q). $^{*}p < 0.05$, $^{**}p < 0.01$, $^{***}p < 0.001$, $^{****}p < 0.0001$.

Figure S5

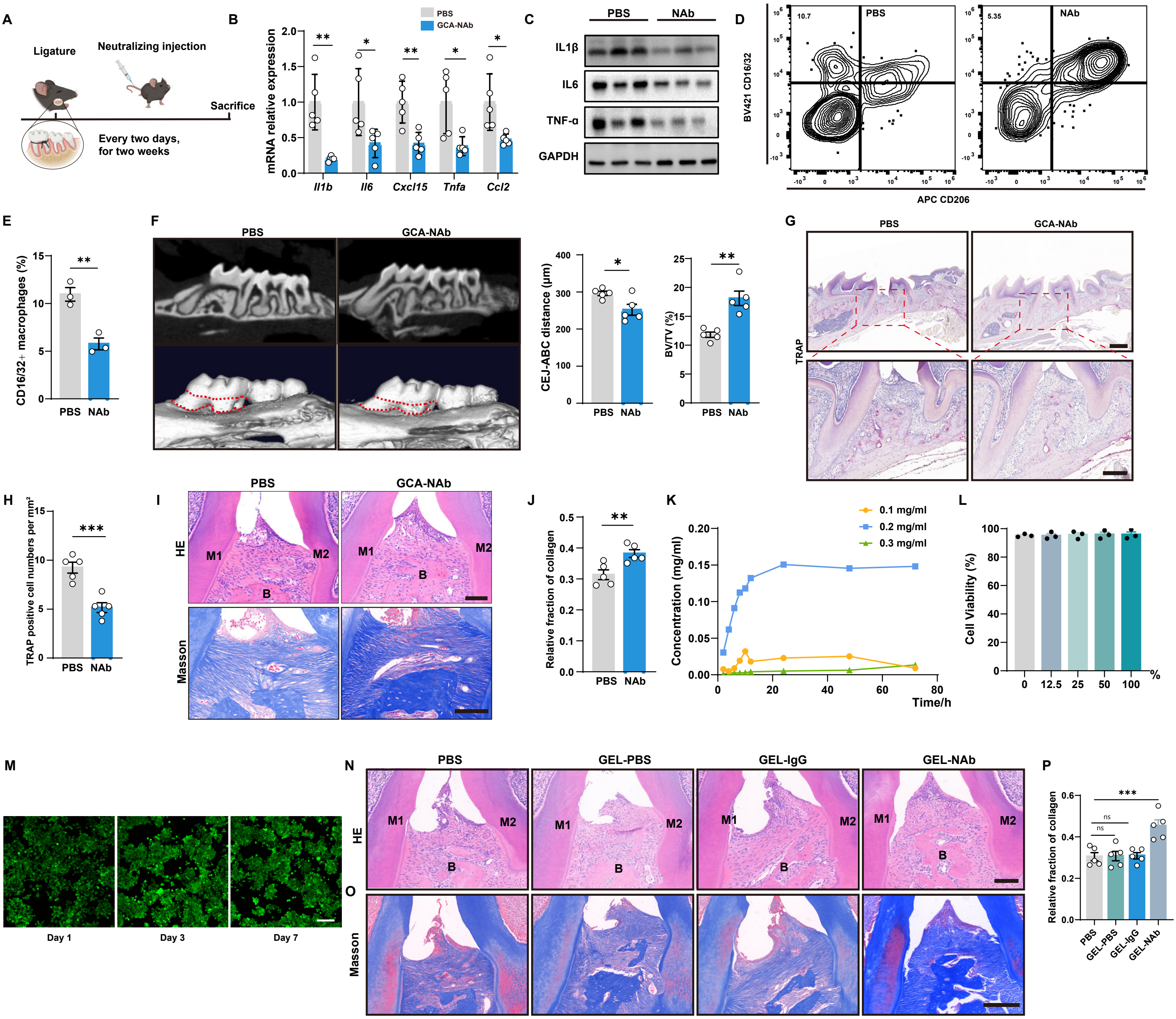

A
Ligature
Neutralizing injection
Sacrifice
Every two days, for two weeks
B
PBS
GCA-NAb
mRNA relative expression
Il1b
Il6
Cxcl15
Tnfa
Ccl2
C
PBS
NAb
IL1β
IL6
TNF-α
GAPDH
D
10.7
PBS
5.35
NAb
BV421 CD16/32
APC CD206
E
CD16/32+ macrophages (%)
PBS
NAb
F
PBS
GCA-NAb
CEJ-ABC distance (μm)
BV/TV (%)
G
PBS
GCA-NAb
TRAP
H
TRAP positive cell numbers per mm²
I
PBS
GCA-NAb
HE
Masson
M1
M2
B
J
Relative fraction of collagen
K
0.1 mg/ml
0.2 mg/ml
0.3 mg/ml
Concentration (mg/ml)
Time/h
L
Cell Viability (%)
M
Day 1
Day 3
Day 7
N
PBS
GEL-PBS
GEL-IgG
GEL-NAb
HE
O
Masson
P
Relative fraction of collagen
ns

Figure S5. Systemic Neutralization of GCA Attenuates Inflammation and Preserves Periodontal Tissues and Bone. (A) Schematic illustration of the ligature-induced periodontitis model and systemic administration of GCA-NAb (1 mg/kg) via tail vein injection every other day for 2 weeks. Created with BioRender.com. (B) RT-qPCR analysis of pro-inflammatory cytokine expression in gingival tissues from PBS- and GCA-NAb-treated (NAb) mice under ligature conditions (n = 5). (C) Representative images of western blot analysis showing protein expression of IL1β, IL6, and TNF-α in gingival tissues from PBS- and GCA-NAb-treated mice (n = 5). (D-E) Flow cytometric analysis (D) and quantification (E) of $CD16/32^{+}$ pro-inflammatory macrophages in gingival tissues (n = 3). (F) Representative micro-CT images and three-dimensional reconstructions of maxillae from PBS- and GCA-NAb-treated mice (left), with quantitative analysis of CEJ-ABC distance and BV/TV ratio (right) based on micro-CT measurements (n = 5). (G-H) Representative TRAP staining images (G) and quantification (H) of TRAP-positive cells per millimeter of alveolar bone surface (scale bar, 500 μm [upper] or 200 μm [lower]; n = 5). (I) Representative H&E staining (upper) and Masson staining (lower) of periodontal tissues from PBS- and GCA-NAb-treated mice (scale bar, 100 μm). (J) The quantification of collagen volume fraction in gingival tissues based on Masson staining (n = 5). (K) Cumulative total protein release from TS-HBC hydrogels containing varying amounts of GCA-neutralizing antibody, quantified by BCA assay. (n = 3). (L) CCK-8 assay evaluating the cytotoxicity of GCA-NAb@TS-HBC hydrogel (0.2 mg/ml) extracts at different dilution ratios (12.5%, 25%, 50%, and 100%) following incubation with RAW264.7 (n = 3). (M) Live/dead staining of RAW264.7 cultured with GCA-NAb@TS-HBC hydrogel (0.2 mg/ml) extracts at 50% dilution ratio for 1, 3, or 7 days. Live cells are shown in green and dead cells in red. (N, O) Representative H&E staining (N) and Masson staining (O) of periodontal tissues from mice treated with PBS, GEL-PBS, GEL-IgG, or GEL-NAb following ligature-induced periodontitis and subsequent local hydrogel injection (scale bar, 100 μm). (P) The quantification of collagen volume fraction in gingival tissues based on Masson

staining (n = 5). Data are shown as the mean ± SEM. n indicates the number of biologically independent samples examined. Statistical significance was determined using two-sided Student's t test (B, E-F, H, J), one-way ANOVA followed by Dunnett's test for comparison with the 0% or PBS group (L, P). *$p < 0.05$, **$p < 0.01$, ***$p < 0.001$, ****$p < 0.0001$.

Table S1. Key anitbodies table

Antibodies	Source	Identifier
Anti-GCA	Abcam	Cat#ab231632; RRID: AB_3716500
Anti-IL-1β	Santa Cruz Biotechnology	Cat#sc-7884; RRID: AB_2124476
Anti-IL-6	Santa Cruz Biotechnology	Cat#sc-57315; RRID: AB_2127596
Anti-TNF-α	Santa Cruz Biotechnology	Cat#sc-52746; RRID: AB_630341
Anti-β-Actin	Proteintech	Cat#66009-1-Ig; RRID: AB_2687938
Anti-GAPDH	Proteintech	Cat#10494-1-AP; RRID: AB_2263076
Anti-Pan-Keratin	Proteintech	Cat#26411-1-AP; RRID: AB_2880505
Anti-FSP1	Proteintech	Cat#16105-1-AP; RRID: AB_11042591
Anti-NF-κB p65	Cell Signaling Technology	Cat#8242; RRID: AB_10859369
Anti-phospho-NF-κB p65	Cell Signaling Technology	Cat#3033; RRID: AB_331284
Anti-Vimentin	Huabio	Cat#ET1610-39; RRID: AB_3069923
Zombie Aqua™ Fixable Viability Kit	BioLegend	Cat#423101; RRID: AB_3716502
APC/Cyanine7 anti-mouse CD45 Antibody	BioLegend	Cat#103116; RRID: AB_312981
PE/Cyanine7 anti-mouse F4/80 Antibody	BioLegend	Cat#123114; RRID: AB_893478
FITC anti-mouse/human CD11b Antibody	BioLegend	Cat#101206; RRID: AB_312789
PE/Cyanine7 anti-mouse F4/80 Antibody	BioLegend	Cat#123114; RRID: AB_893478
Brilliant Violet 421™ anti-mouse CD16/32 Antibody	BioLegend	Cat#101332; RRID: AB_2650889
APC anti-mouse CD206 (MMR) Antibody	BioLegend	Cat#141708; RRID: AB_10900231
PE/Cyanine7 anti-mouse Ly-6C Antibody	BioLegend	Cat#128018; RRID: AB_1732082
Pacific BlueTM anti-mouse Ly-6G	BioLegend	Cat#127612; RRID: AB_2251161
Anti-F4/80	Abcam	Cat#ab6640; RRID: AB_1140040
DYKDDDDK tag Polyclonal antibody (Binds to FLAG® tag epitope)	Proteintech	Cat#20543-1-AP; RRID: AB_11232216
MYC tag Monoclonal antibody	Proteintech	Cat#60003-2-Ig; RRID: AB_2734122
His-Tag Monoclonal antibody	Proteintech	Cat#66005-1-Ig; RRID: AB_11232599
HA tag Polyclonal antibody	Proteintech	Cat#51064-2-AP; RRID: AB_11042321
MYH9 Polyclonal antibody	Proteintech	Cat#11128-1-AP; RRID: AB_2147294
Phospho-Myosin IIa (Ser1943) Antibody	Cell Signaling Technology	Cat#5026S; RRID: AB_10576567
Lamin B1 Polyclonal antibody	Proteintech	Cat#12987-1-AP; RRID: AB_2136290

Table S2. Nucleotide sequences of primers used for quantitative RT-PCR detection.

Gene	Primer	Sequence
Human-GCA	Forward primer	TGCATTCAAAGAGCTATGGGC
	Reverse primer	CTGTGCCACTTCCATCTTGAT
Human-IL1B	Forward primer	ACAGATGAAGTGCTCCTTCCA
	Reverse primer	GTCGGAGATTCGTAGCTGGAT
Human-IL6	Forward primer	GGTACATCCTCGACGGCATCT
	Reverse primer	GTGCCTCTTTGCTGCTTTC
Human-CXCL8	Forward primer	CACCGGAAGGAACCATCTCA
	Reverse primer	AGAGCCACGGCCAGCTT
Human-TNF	Forward primer	CTCTTCTGCCTGCTGCACTTTG
	Reverse primer	ATGGGCTACAGGCTTGTCACTC
Human-CCL2	Forward primer	GCATCCACGTGTTGGCTCA
	Reverse primer	CTCCAGCCTACTCATTGGGATCA
Human-IL4	Forward primer	GCCTGGCGGGCTTGA
	Reverse primer	AGCCTTTCCAAGAAGTTTTCCA
Human-TNFRSF11B	Forward primer	CAACACAGCTCACAAGAACAG
	Reverse primer	GAAGGTGAGGTTAGCATGTCC
Human-CD44	Forward primer	TGACACTGTCCAAAGGTTTTC
	Reverse primer	TCACTAATAGGGCCAGCCTC
Human-MYH9	Forward primer	CCTGCCATAAGGGAACCTAATCAC
	Reverse primer	GCGCTCTGGTGCCTCTCCTA
Human-GAPDH	Forward primer	CGGAGTCAACGGATTTGGTCGTAT
	Reverse primer	AGCCTTCTCCATGGTGGTGAAGAC
Mouse-Gca	Forward primer	GGGGCGTTTGGAAACTTCAG
	Reverse primer	AGGGGAGTAGCTGTCAGAATAAC
Mouse-Il1b	Forward primer	CCCTGCAGCTGGAGAGTGTGGA
	Reverse primer	CTGAGCGACCTGTCTTGGCCG
Mouse-Il6	Forward primer	CAAAGCCAGAGTCCTTCAGAG
	Reverse primer	AGCATTGGAAATTGGGGTAG
Mouse-Cxcl15	Forward primer	CTGGTCCATGCTCCTGCTG
	Reverse primer	GGACGGACGAAGATGCCTAG
Mouse-Tnf	Forward primer	TCTTCTCATTCCTGCTTGTGG
	Reverse primer	ATGAGAGGGAGGCCATTTG
Mouse-Ccl2	Forward primer	TTAAAAACCTGGATCGGAACCAA
	Reverse primer	GCATTAGCTTCAGATTTACGGGT
Mouse-Ctsk	Forward primer	GGATGAAATCTCTCGGCGTTT
	Reverse primer	GGTTATGGGCAGAGATTTGCTT
Mouse-Nfatc1	Forward primer	CCTTCGGAAGGGTGCCTTTT
	Reverse primer	CGGCTGCCTTCCGTCTCATAG
