# Supplementary material for: Myeloid‐Derived Grancalcin Promotes Periodontal Inflammation and Pathological Bone Remodeling in Periodontitis
Source: Adv Sci (Weinh). 2026 Mar 25;13(31):e19267. doi: 10.1002/advs.202519267 (PMC13588070; doi:10.1002/advs.202519267)
Supplement: Supplementary file 2 [file ADVS-13-e19267-s001.zip › Supplementary Data_microscope images.pdf]

Figure 1

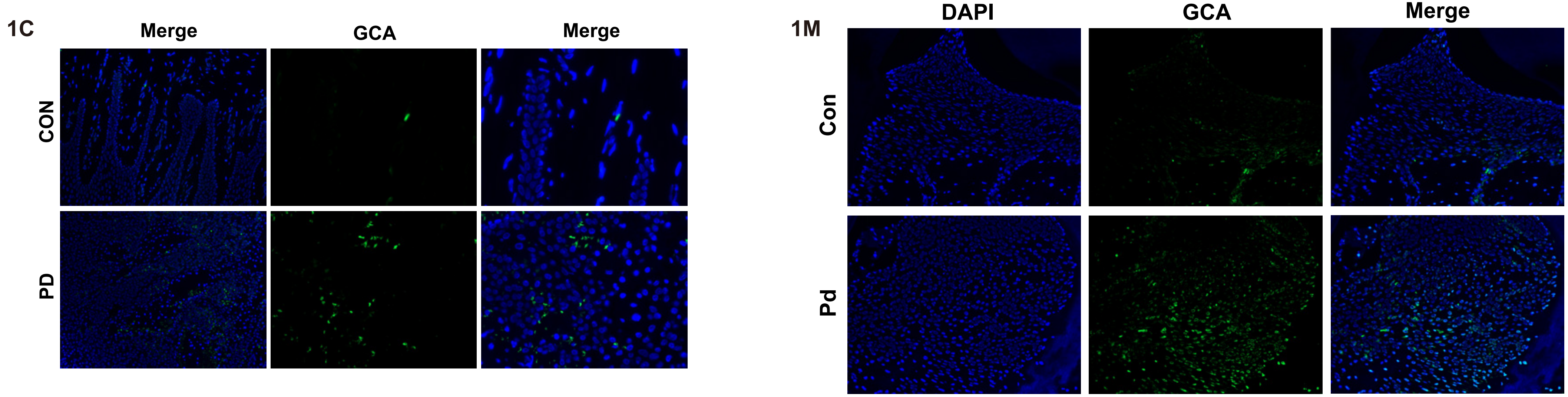

Figure 2

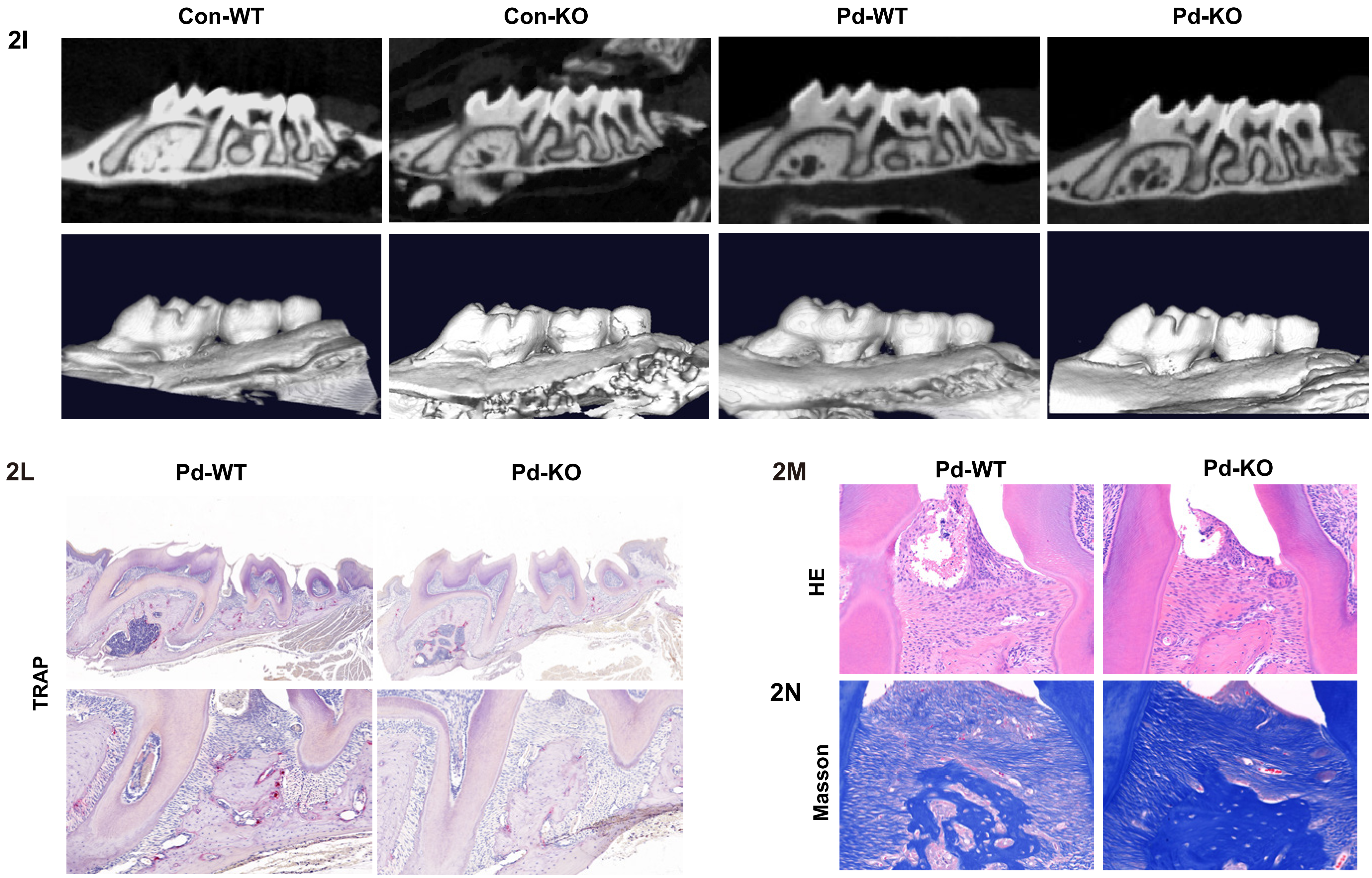
2I
Con-WT
Con-KO
Pd-WT
Pd-KO
2L
Pd-WT
Pd-KO
TRAP
2M
Pd-WT
Pd-KO
HE
2N
Masson

Figure 3

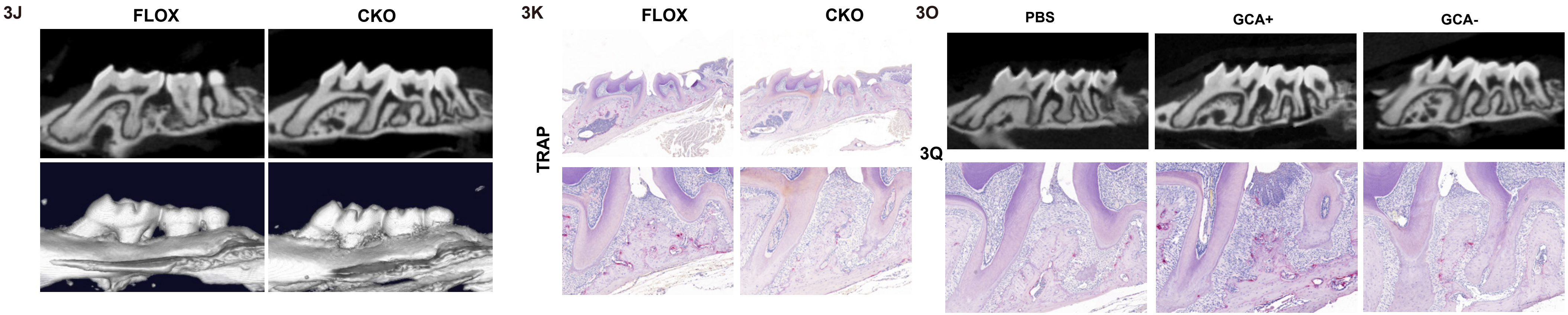

Figure 4

4K

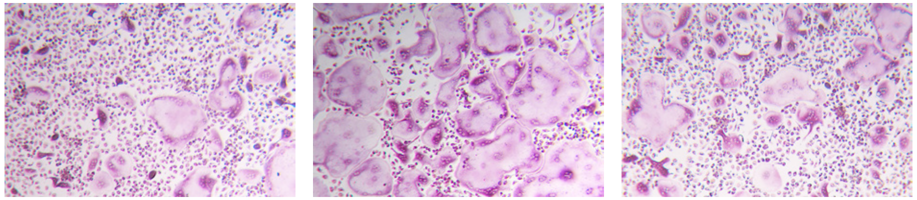

4P

4R

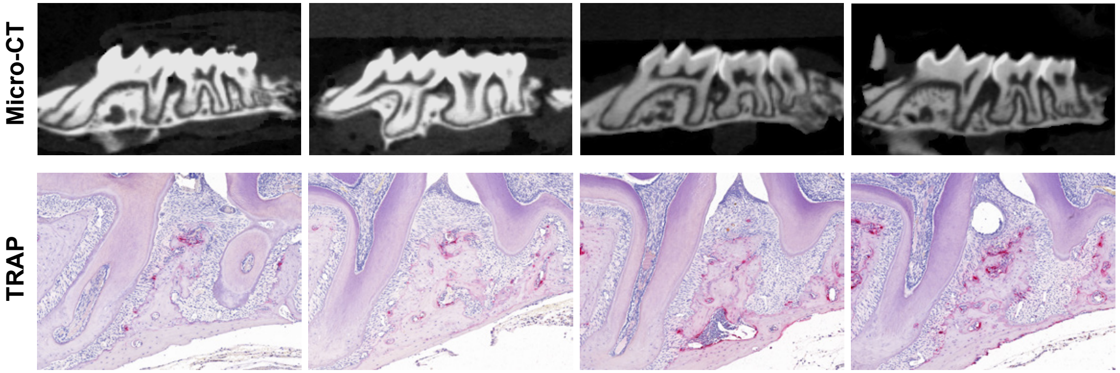

Figure 5

5I

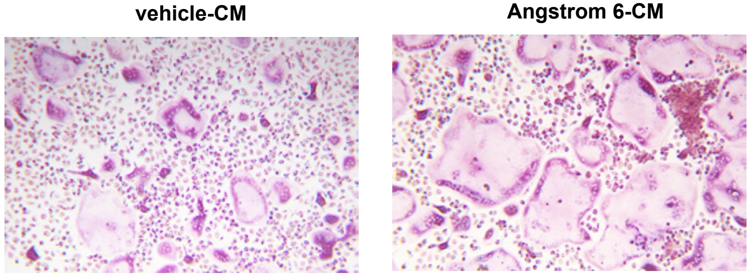

5P

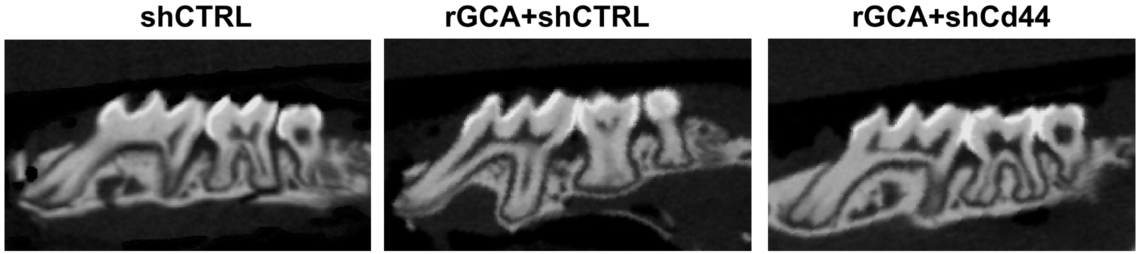

5R

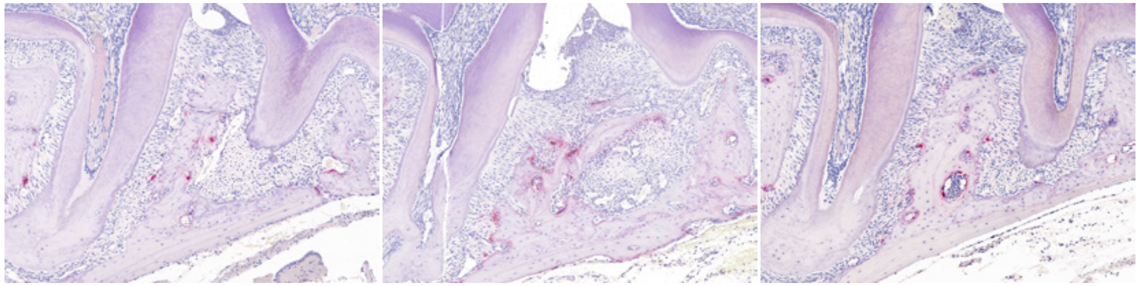

Figure 6

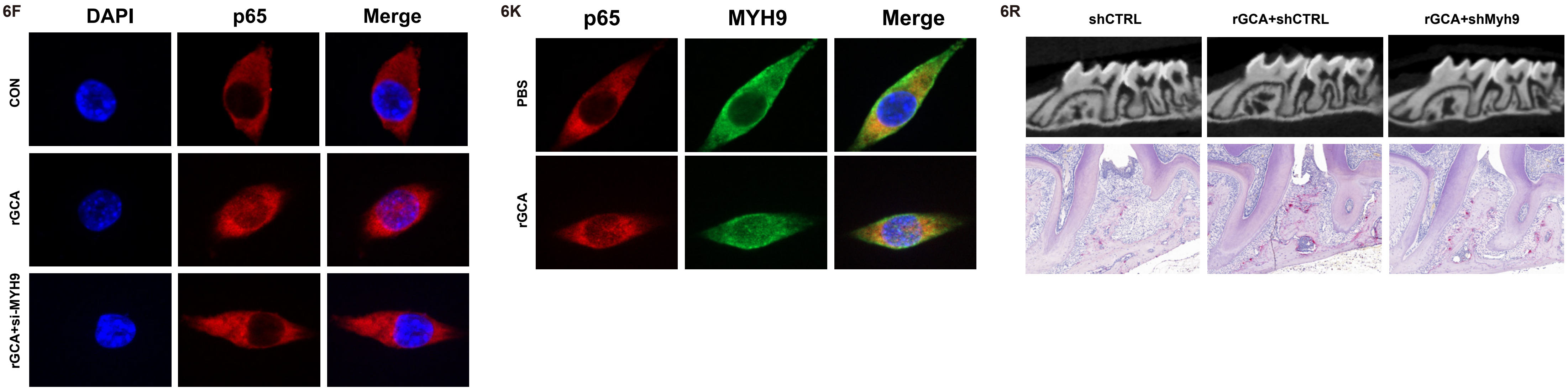
6F
DAPI
p65
Merge
CON
rGCA
rGCA+si-MYH9
6K
p65
MYH9
Merge
PBS
rGCA
6R
shCTRL
rGCA+shCTRL
rGCA+shMyh9

Figure 7

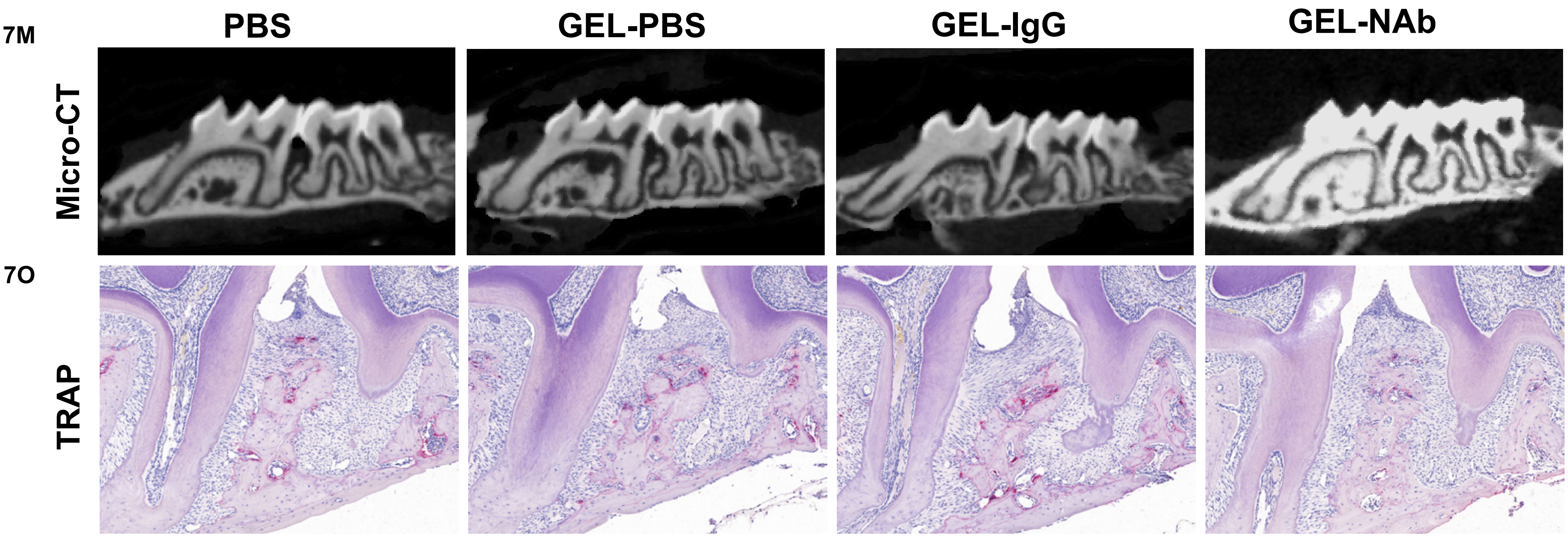

Figure S1

S1B

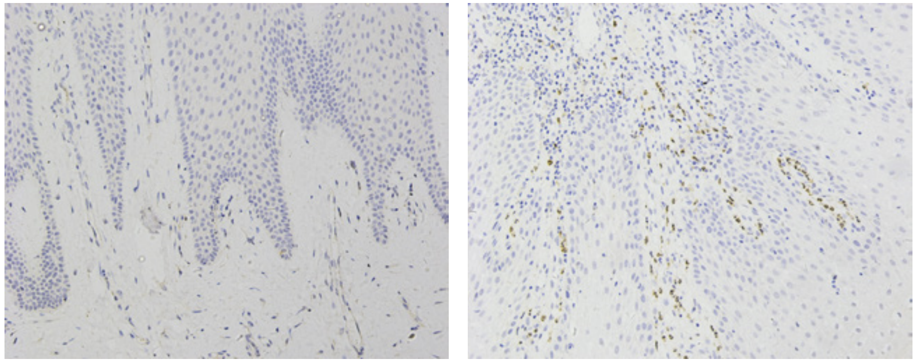

S1I

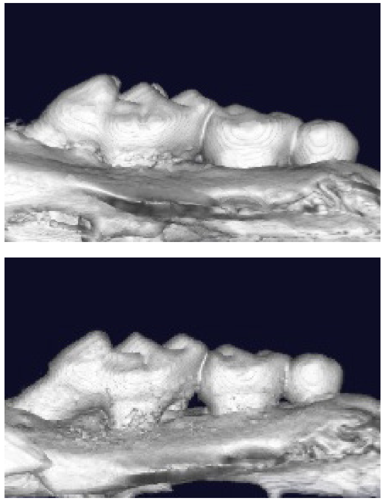

S1K

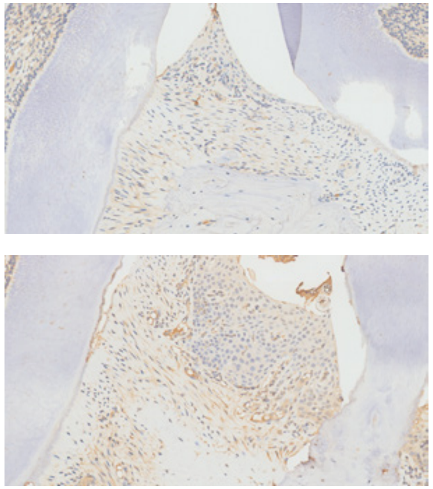

Figure S3

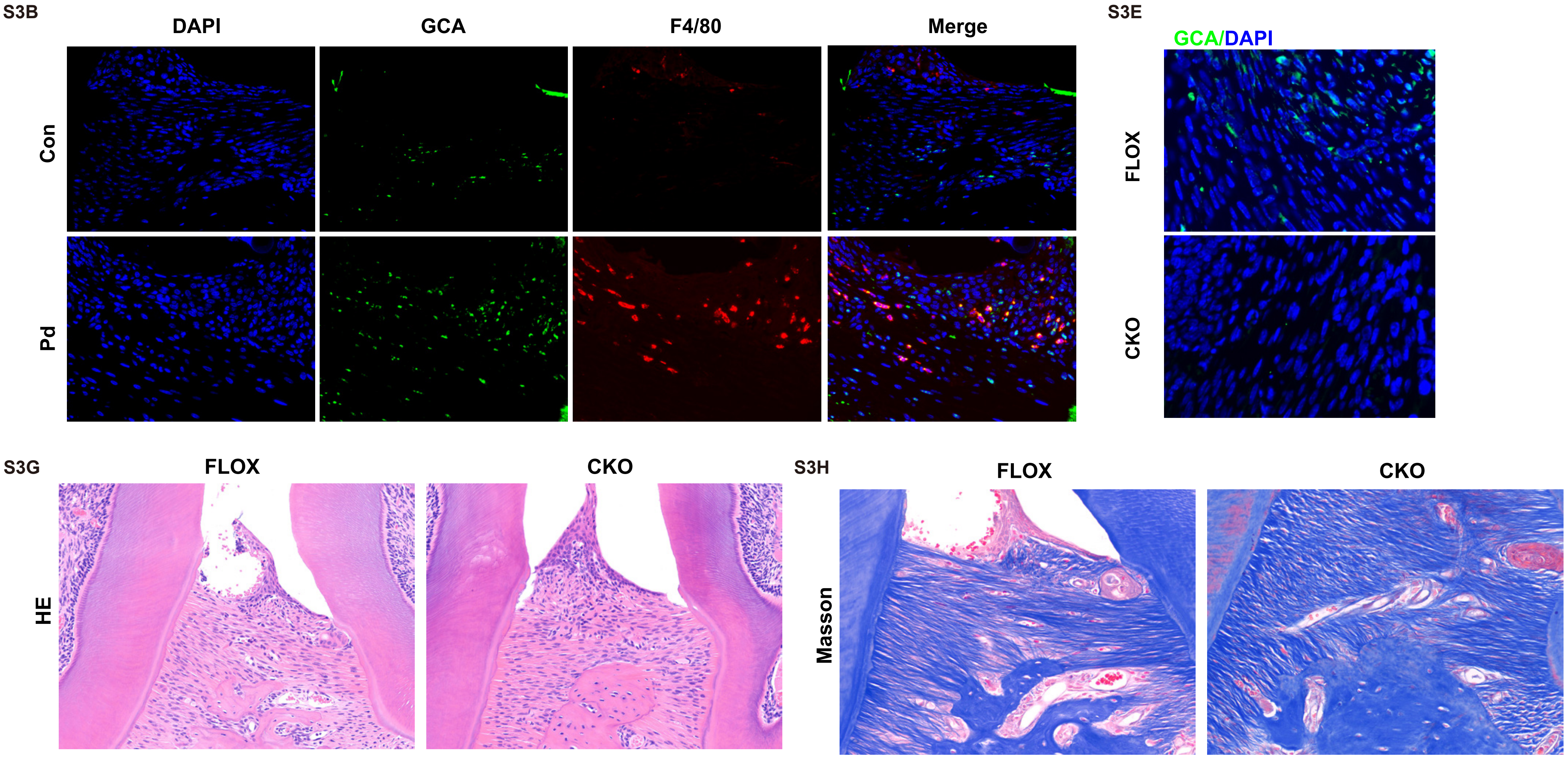
S3B
DAPI
GCA
F4/80
Merge
Con
Pd
S3E
GCA/DAPI
FLOX
CKO
S3G
FLOX
CKO
HE
S3H
FLOX
CKO
Masson

Figure S4

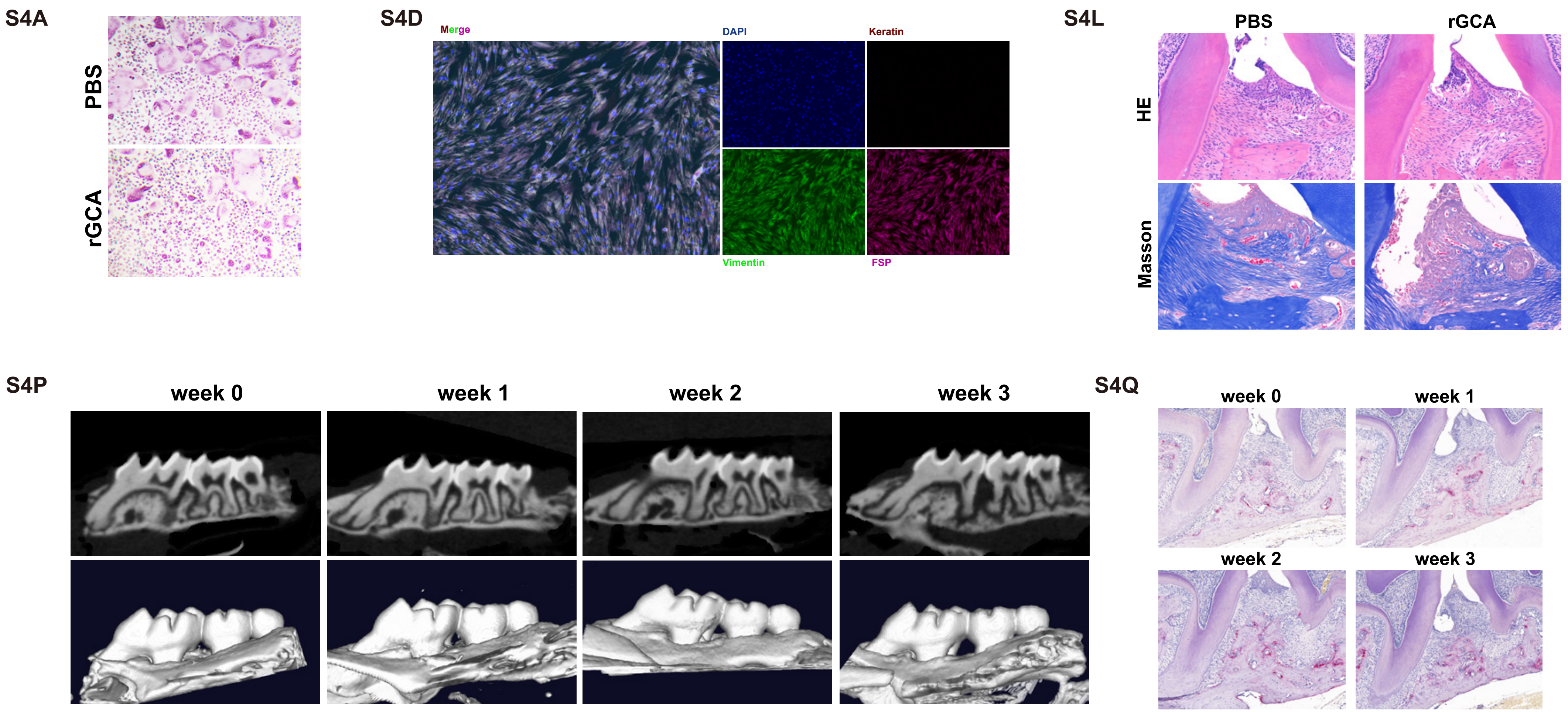
S4A
PBS
rGCA
S4D
Merge
DAPI
Keratin
Vimentin
FSP
S4L
PBS
rGCA
HE
Masson
S4P
week 0
week 1
week 2
week 3
S4Q
week 0
week 1
week 2
week 3

Figure S5

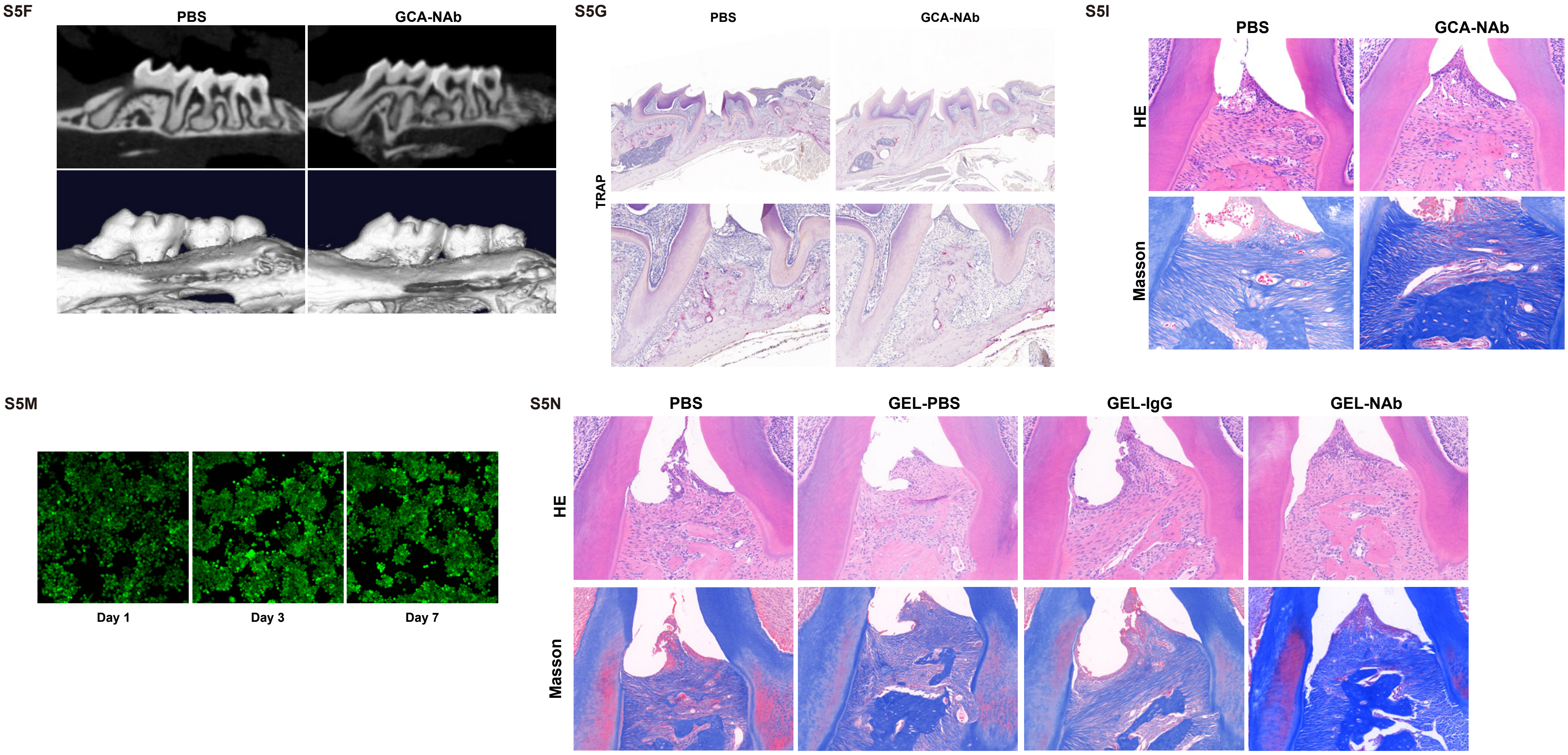

S5F
PBS
GCA-NAb
S5G
PBS
GCA-NAb
TRAP
S5I
PBS
GCA-NAb
HE
Masson
S5M
Day 1
Day 3
Day 7
S5N
PBS
GEL-PBS
GEL-IgG
GEL-NAb
HE
Masson
