# Supplementary material for: Myeloid‐Derived Grancalcin Promotes Periodontal Inflammation and Pathological Bone Remodeling in Periodontitis
Source: Adv Sci (Weinh). 2026 Mar 25;13(31):e19267. doi: 10.1002/advs.202519267 (PMC13588070; doi:10.1002/advs.202519267)

Figure 1

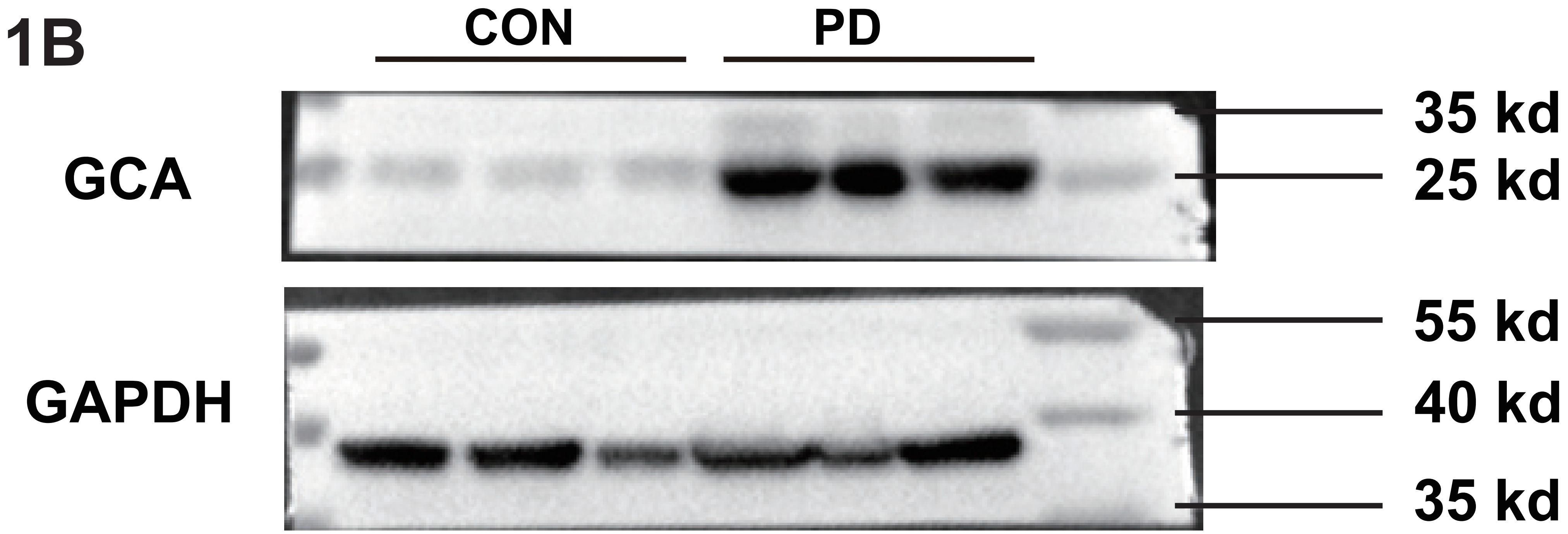

Figure 2

2G

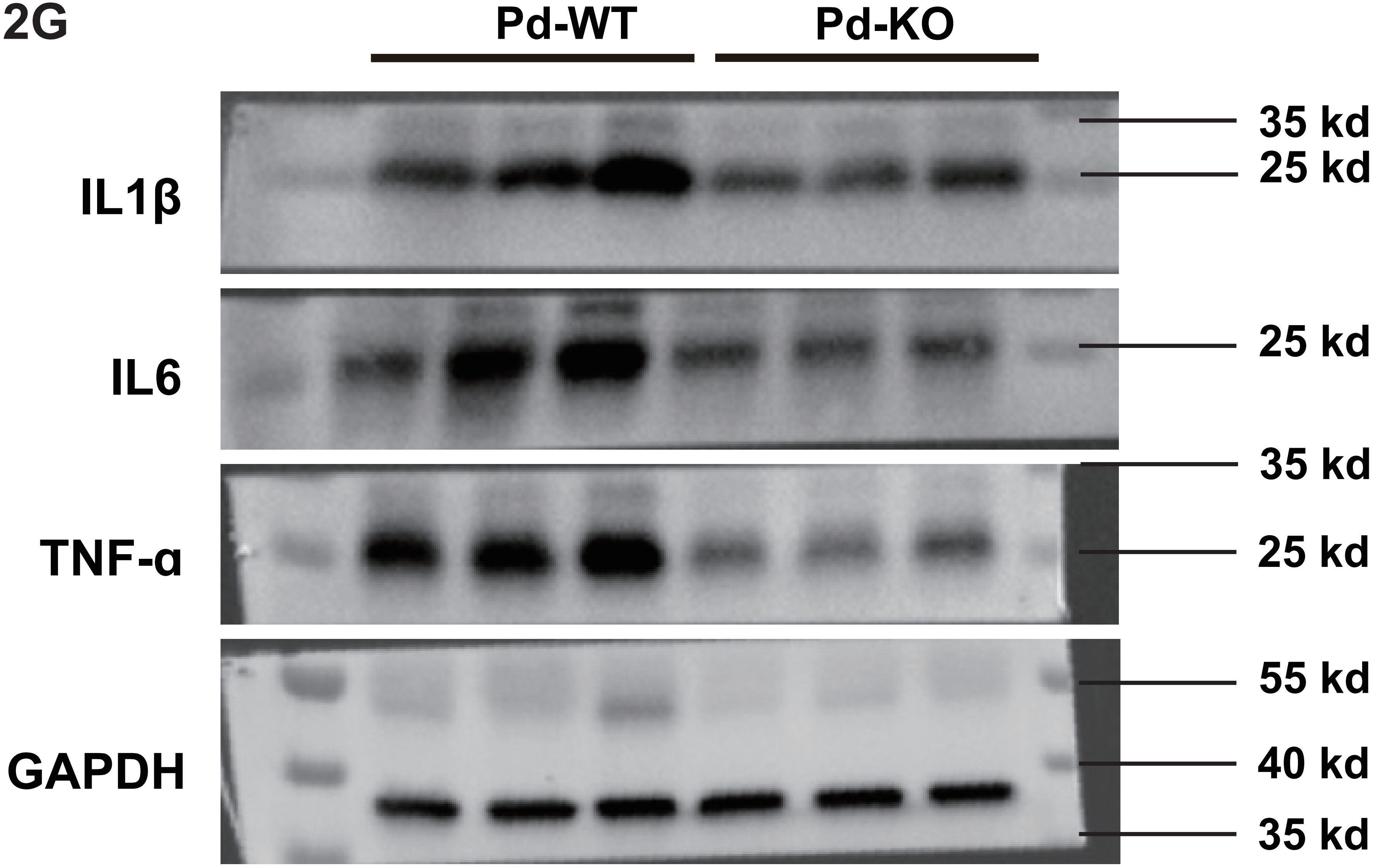
Pd-WT
Pd-KO
IL1β
IL6
TNF-α
GAPDH
35 kd
25 kd
25 kd
35 kd
25 kd
55 kd
40 kd
35 kd

Figure 3

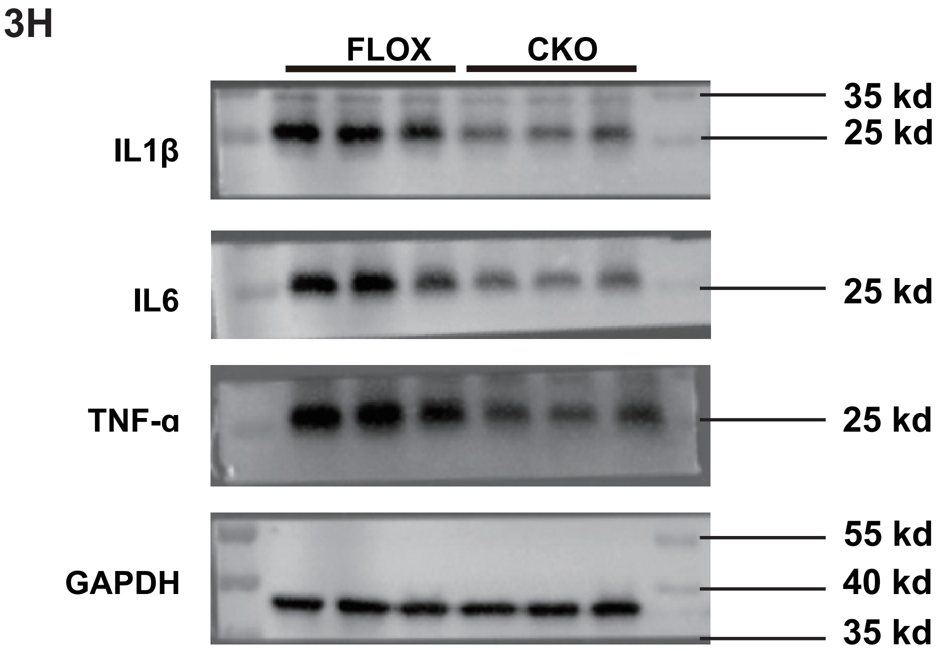
3H
FLOX
CKO
IL1β
IL6
TNF-α
GAPDH
35 kd
25 kd
25 kd
25 kd
55 kd
40 kd
35 kd

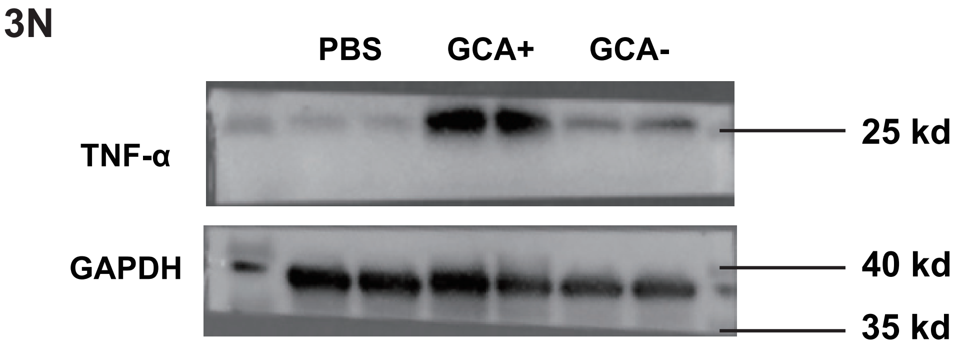
3N
PBS
GCA+
GCA-
TNF-α
GAPDH
25 kd
40 kd
35 kd

Figure 4

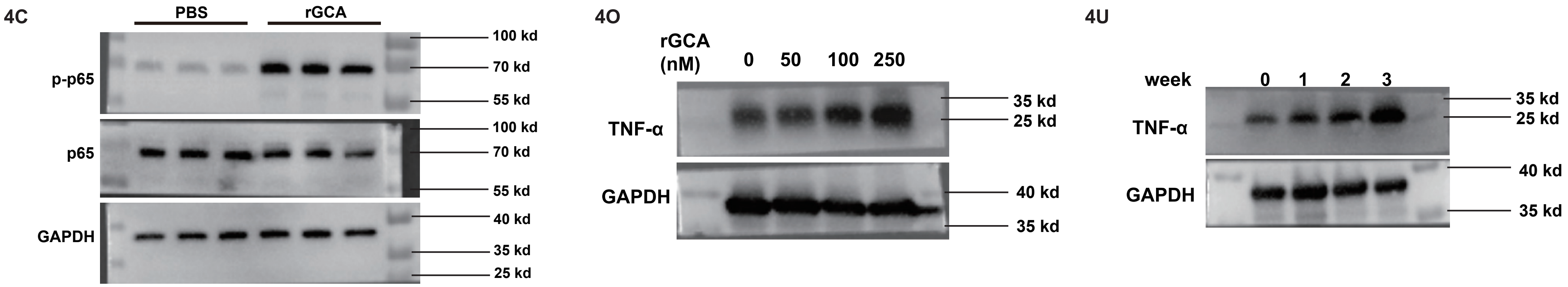
4C
PBS
rGCA
p-p65
p65
GAPDH
100 kd
70 kd
55 kd
100 kd
70 kd
55 kd
40 kd
35 kd
25 kd
4O
rGCA
(nM)
0
50
100
250
TNF-α
GAPDH
35 kd
25 kd
40 kd
35 kd
4U
week
0
1
2
3
TNF-α
GAPDH
35 kd
25 kd
40 kd
35 kd

Figure 5

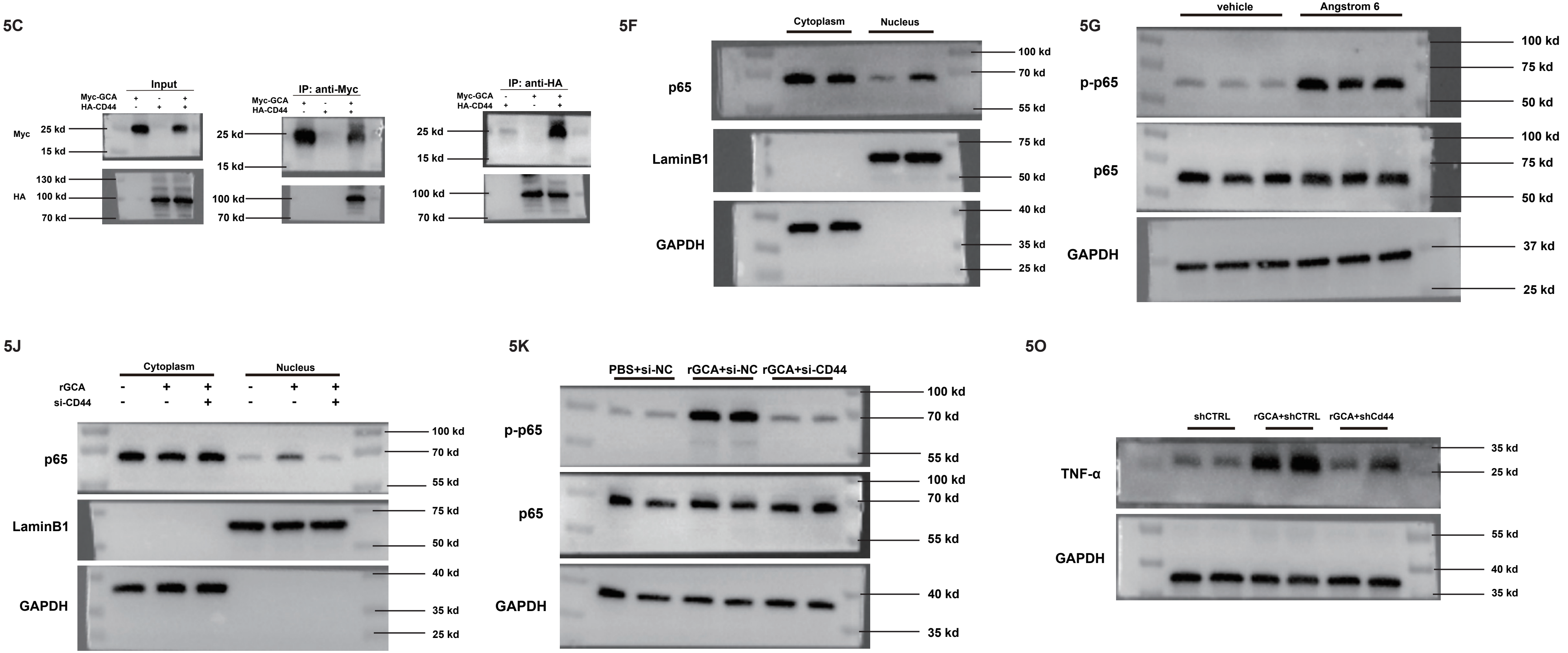
5C
Input
Myc-GCA + - +
HA-CD44 - + +
Myc
25 kd
15 kd
HA
130 kd
100 kd
70 kd
IP: anti-Myc
Myc-GCA + - +
HA-CD44 - + +
25 kd
15 kd
100 kd
70 kd
IP: anti-HA
Myc-GCA - + +
HA-CD44 + - +
25 kd
15 kd
100 kd
70 kd
5F
Cytoplasm
Nucleus
p65
100 kd
70 kd
55 kd
LaminB1
75 kd
50 kd
GAPDH
40 kd
35 kd
25 kd
5G
vehicle
Angstrom 6
p-p65
100 kd
75 kd
50 kd
p65
100 kd
75 kd
50 kd
GAPDH
37 kd
25 kd
5J
Cytoplasm
Nucleus
rGCA - + + - + +
si-CD44 - - + - - +
p65
100 kd
70 kd
55 kd
LaminB1
75 kd
50 kd
GAPDH
40 kd
35 kd
25 kd
5K
PBS+si-NC
rGCA+si-NC
rGCA+si-CD44
p-p65
100 kd
70 kd
55 kd
p65
100 kd
70 kd
55 kd
GAPDH
40 kd
35 kd
5O
shCTRL
rGCA+shCTRL
rGCA+shCd44
TNF-α
35 kd
25 kd
GAPDH
55 kd
40 kd
35 kd

Figure 6

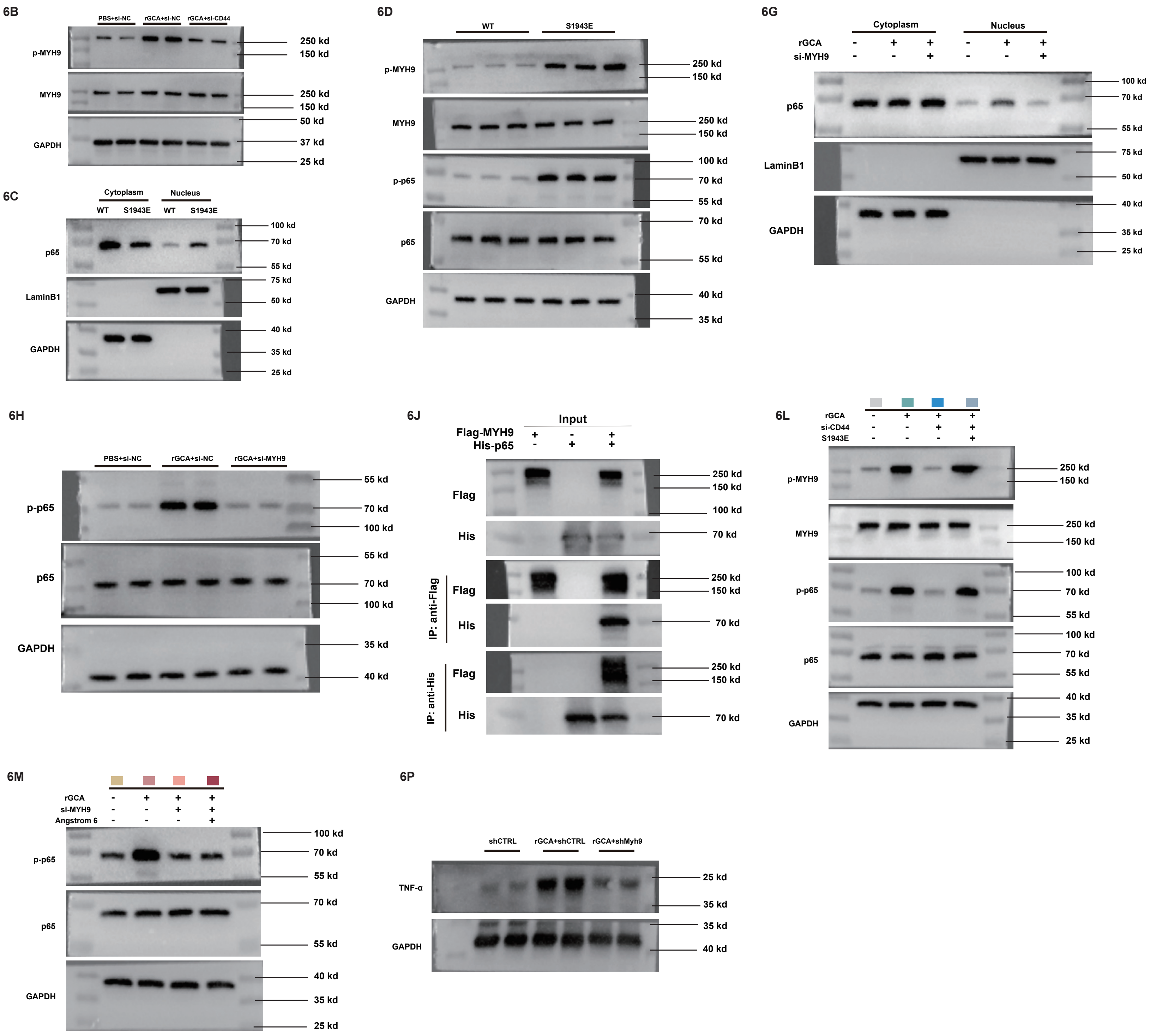
6B
PBS+si-NC
rGCA+si-NC
rGCA+si-CD44
p-MYH9
MYH9
GAPDH
250 kd
150 kd
250 kd
150 kd
50 kd
37 kd
25 kd
6C
Cytoplasm
Nucleus
WT
S1943E
WT
S1943E
p65
LaminB1
GAPDH
100 kd
70 kd
55 kd
75 kd
50 kd
40 kd
35 kd
25 kd
6D
WT
S1943E
p-MYH9
MYH9
p-p65
p65
GAPDH
250 kd
150 kd
250 kd
150 kd
100 kd
70 kd
55 kd
70 kd
55 kd
40 kd
35 kd
6G
Cytoplasm
Nucleus
rGCA
si-MYH9
p65
LaminB1
GAPDH
100 kd
70 kd
55 kd
75 kd
50 kd
40 kd
35 kd
25 kd
6H
PBS+si-NC
rGCA+si-NC
rGCA+si-MYH9
p-p65
p65
GAPDH
55 kd
70 kd
100 kd
55 kd
70 kd
100 kd
35 kd
40 kd
6J
Input
Flag-MYH9
His-p65
Flag
His
IP: anti-Flag
Flag
His
IP: anti-His
Flag
His
250 kd
150 kd
100 kd
70 kd
250 kd
150 kd
70 kd
250 kd
150 kd
70 kd
6L
rGCA
si-CD44
S1943E
p-MYH9
MYH9
p-p65
p65
GAPDH
250 kd
150 kd
250 kd
150 kd
100 kd
70 kd
55 kd
100 kd
70 kd
55 kd
40 kd
35 kd
25 kd
6M
rGCA
si-MYH9
Angstrom 6
p-p65
p65
GAPDH
100 kd
70 kd
55 kd
70 kd
55 kd
40 kd
35 kd
25 kd
6P
shCTRL
rGCA+shCTRL
rGCA+shMyh9
TNF-α
GAPDH
25 kd
35 kd
35 kd
40 kd

Figure 7

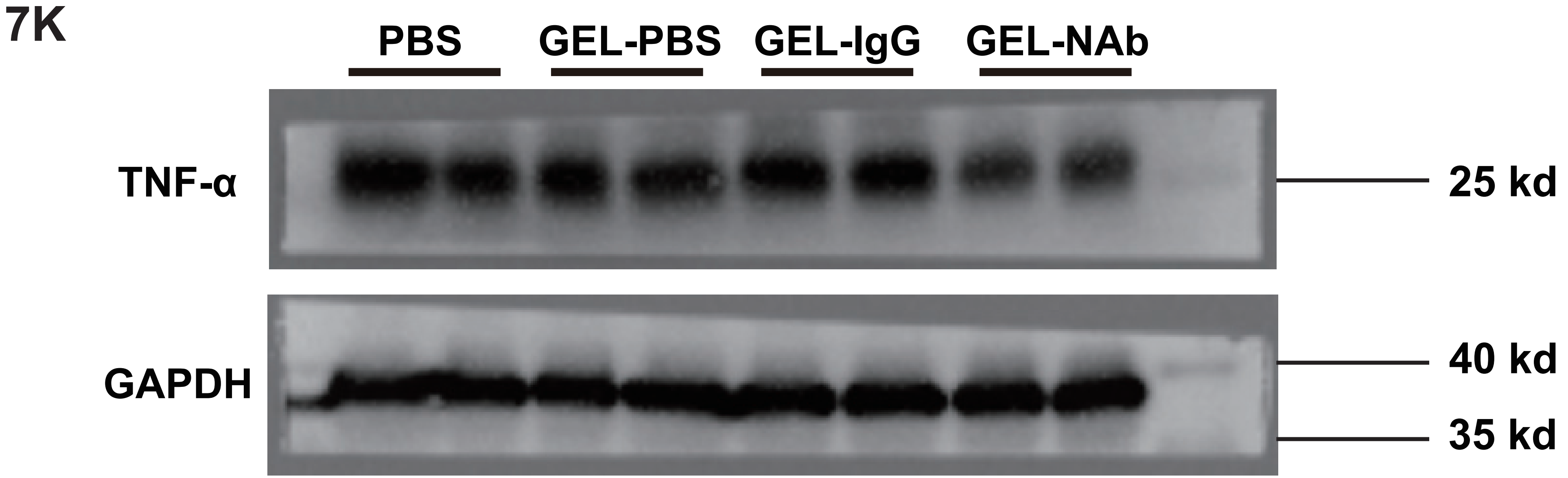

Figure S3

S3D

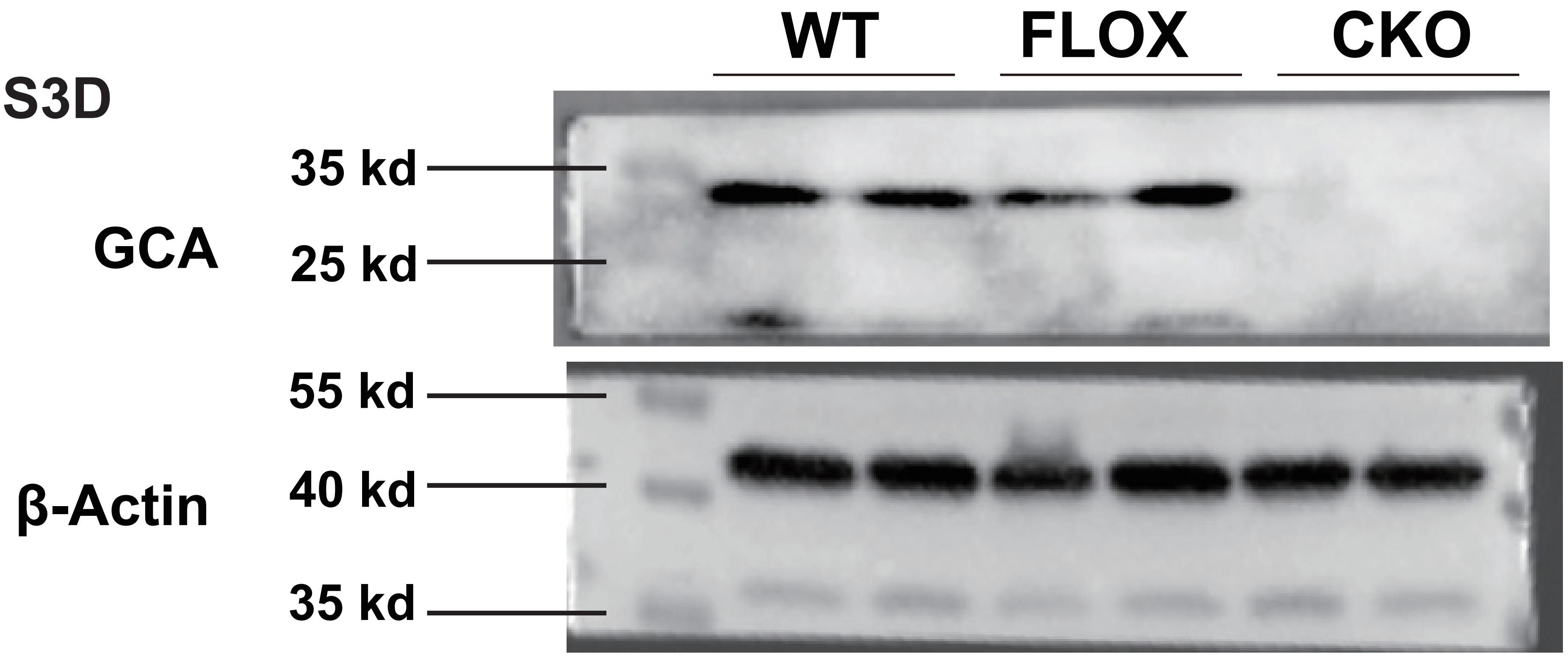

Figure S5

S5C

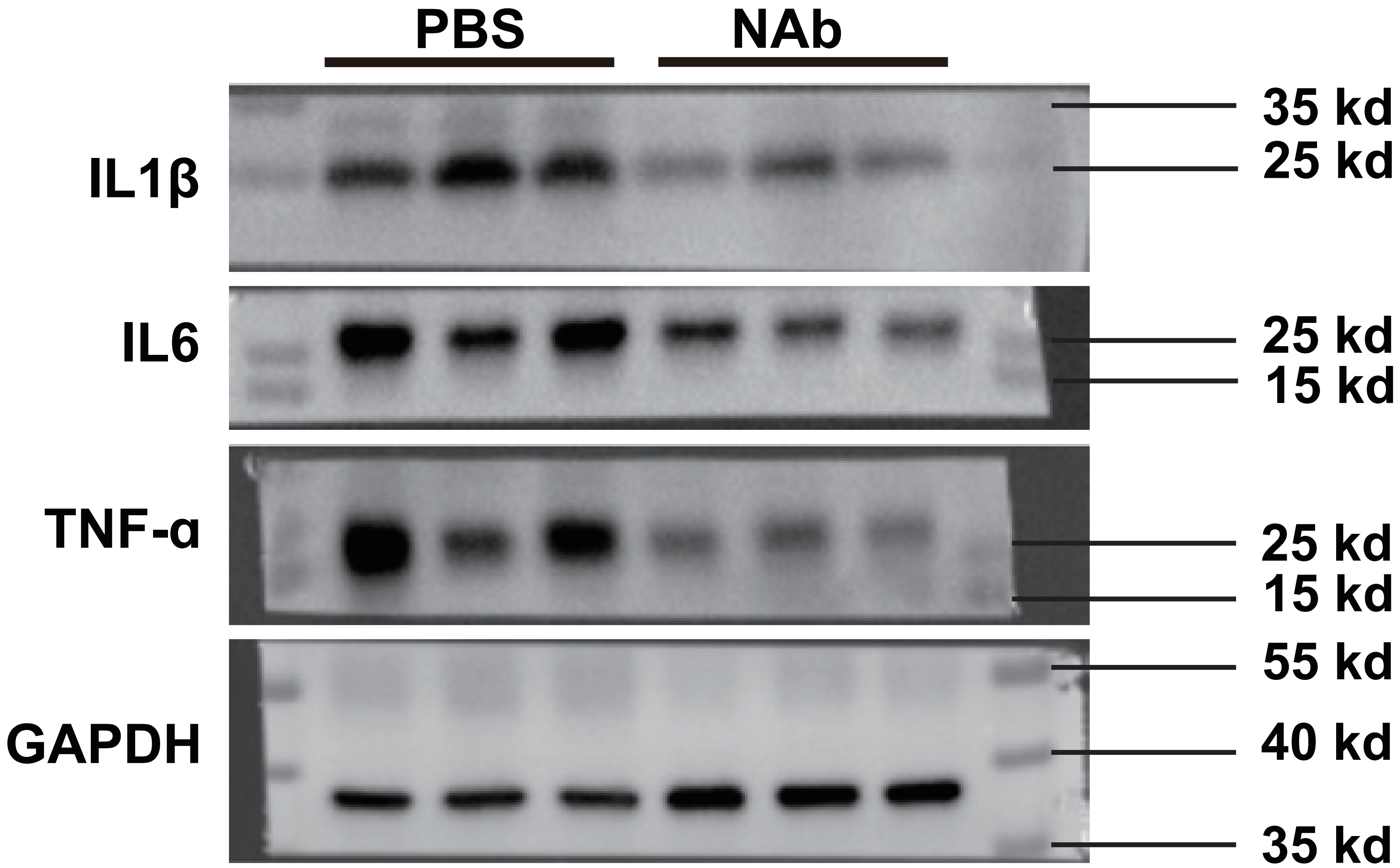

Supplement: Supplementary file 2 [file ADVS-13-e19267-s001.zip › Supplementary Data_blots.pdf]
